# NF-**κ**B drives epithelial-mesenchymal mechanisms of lung fibrosis in a translational lung cell model

**DOI:** 10.1172/jci.insight.154719

**Published:** 2023-02-08

**Authors:** Patrick Sieber, Anny Schäfer, Raphael Lieberherr, Silvia L. Caimi, Urs Lüthi, Jesper Ryge, Jan H. Bergmann, François Le Goff, Manuel Stritt, Peter Blattmann, Bérengère Renault, Patrick Rammelt, Bruno Sempere, Diego Freti, Rolf Studer, Eric S. White, Magdalena Birker-Robaczewska, Maxime Boucher, Oliver Nayler

**Affiliations:** 1 Idorsia Pharmaceuticals Ltd., Allschwil, Switzerland.; 2 Division of Pulmonary and Critical Care Medicine, Department of Internal Medicine, University of Michigan Medical School, Ann Arbor, Michigan, USA.

**Keywords:** Cell Biology, Pulmonology, Fibrosis

## Abstract

In the progression phase of idiopathic pulmonary fibrosis (IPF), the normal alveolar structure of the lung is lost and replaced by remodeled fibrotic tissue and by bronchiolized cystic airspaces. Although these are characteristic features of IPF, knowledge of specific interactions between these pathological processes is limited. Here, the interaction of lung epithelial and lung mesenchymal cells was investigated in a coculture model of human primary airway epithelial cells (EC) and lung fibroblasts (FB). Single-cell RNA sequencing revealed that the starting EC population was heterogenous and enriched for cells with a basal cell signature. Furthermore, fractions of the initial EC and FB populations adopted distinct pro-fibrotic cell differentiation states upon cocultivation, resembling specific cell populations that were previously identified in lungs of patients with IPF. Transcriptomic analysis revealed active NF-κB signaling early in the cocultured EC and FB, and the identified NF-κB expression signatures were found in “HAS1 High FB” and “PLIN2+ FB” populations from IPF patient lungs. Pharmacological blockade of NF-κB signaling attenuated specific phenotypic changes of EC and prevented FB-mediated interleukin-6, interleukin-8, and CXC chemokine ligand 6 cytokine secretion, as well as collagen α-1(I) chain and α–smooth muscle actin accumulation. Thus, we identified NF-κB as a potential mediator, linking epithelial pathobiology with fibrogenesis.

## Graphical abstract

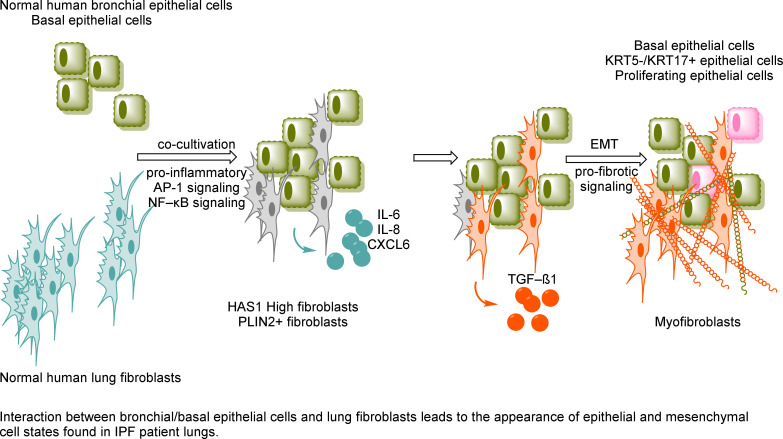

## Introduction

Idiopathic pulmonary fibrosis (IPF), the most severe form of pulmonary fibrosis, is a chronic progressive disease leading to respiratory failure and death or lung transplantation with a median survival of 6–8 years from diagnosis. IPF is an incurable disease and currently available treatments can only slow the rate of decline of forced vital capacity of the lung (1). The disease is characterized by pathological epithelial remodeling, and accumulation of extracellular matrix (ECM) in the peripheral lung, accompanied by the destruction of functional alveolar gas exchange structures (2).

Disease progression may involve continuous proliferation of dysfunctional reprogrammed bronchoalveolar epithelial cells (EC) (1). The timing and nature of triggering and subsequent reprogramming of lung EC, however, need further exploration (1, 3). Recent studies using single-cell RNA sequencing (sc-RNA-Seq) have identified specific cell populations that were enriched in patients with IPF compared with control lungs (4–8). While EC play a major role in the formation of bronchiolized cystic airspaces, myofibroblasts and related mesenchymal cells are mainly responsible for the synthesis of ECM components and fibrotic distortion of the lung. To improve our understanding of the pathological epithelial-mesenchymal interaction, we set out to model the “epithelial-mesenchymal module” in vitro by coculturing EC and fibroblasts (FB). EC and mesenchymal cells have previously been cultured together, resulting in various outcomes that can be attributed to the particular cell types and applied cell culture protocol but also to the frequently added pro-fibrotic stimuli (9–17).

Here, we identified and optimized in vitro conditions that reproducibly recapitulate, chronologically phased and in the absence of added artificial stimuli, a pro-fibrotic phenotype shift in the cultured cells. Upon direct EC/FB contact, coculture of primary normal human lung fibroblasts (NHLF) and normal human bronchiolar epithelial cells (NHBE) led to the robust activation of a fibrogenic program in the cocultivated FB. FB responded with a strong initial inflammatory response, followed by TGF-β–mediated synthesis of ECM components — i.e., collagen α-1(I) chain (COL1A1) — and increased α–smooth muscle actin (α-SMA), which are considered hallmarks of FB-to-myofibroblast transition. Preceding myofibroblast differentiation, coculture induced activation of NF-κB and activator protein 1 (AP-1), with associated downstream gene expression changes, evident already after 3 hours in FB and EC. Using sc-RNA-Seq and genomic data analysis, we show that cocultured EC and FB progressed to cell phenotypes that are also enriched in patients with IPF, and we identified expression of NF-κB and AP-1 gene modules in distinct cell populations of patients with IPF, highlighting the translational potential of our model.

Pharmacological inhibition of NF-κB and AP-1 signaling revealed their driving role in the early stages of fibrogenesis in the coculture model, suggesting that NF-κB and AP-1 signaling might also contribute to the pathobiological process in IPF and could be a potential target for pharmacological intervention.

## Results

### EC induce FB-to-myofibroblast transformation.

**Figure 1:**
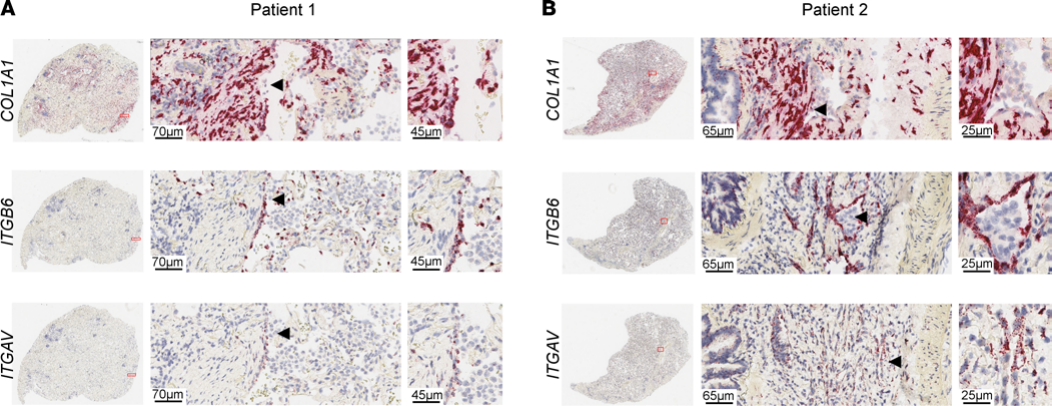
Epithelial and mesenchymal cells are localized in close proximity in lung sections of patients with IPF. Expression of the mesenchymal marker gene *COL1A1*, as well as the epithelial marker genes *ITGB6* and *ITGAV*, as detected by RNAscope ISH on formalin-fixed, paraffin-embedded lung sections of IPF patient 1 (**A**) and 2 (**B**). Boxed area in the low-magnification lung section overviews in the left image indicates the enlarged region. The arrowheads point to the approximate same location in close consecutive tissue sections. Representative images of 2 out of 3 patients with IPF are shown.

Previously, we identified common genomic signatures between samples from IPF patient lungs and bleomycin-treated rat lungs (18). To expand on this work, we investigated expression of selected genes on consecutive histological sections prepared from lung biopsies of patients with IPF and non-IPF control lungs, using RNAscope in situ hybridization (ISH). Areas with detectable gene expression exhibited distribution patterns that were generally more abundant in patients (Supplemental Figures 1 and 2; supplemental material available online with this article; https://doi.org/10.1172/jci.insight.154719DS1). Cells expressing the epithelial markers integrin subunit β6 (*ITGB6*) and integrin subunit αV (*ITGAV*) overlaid foci with high *COL1A1* expression in lung regions considered to have active disease (Figure 1 and Supplemental Figure 2). In particular, *ITGB6* and *COL1A1* expression was detected in cells that were in very close proximity to each other but appeared to be mutually exclusive (Figure 1). Collectively, we found that EC and FB/myofibroblasts appeared in very close contact in areas of active disease in IPF patient lungs, which is in accordance with published data (4–6).

**Figure 2:**
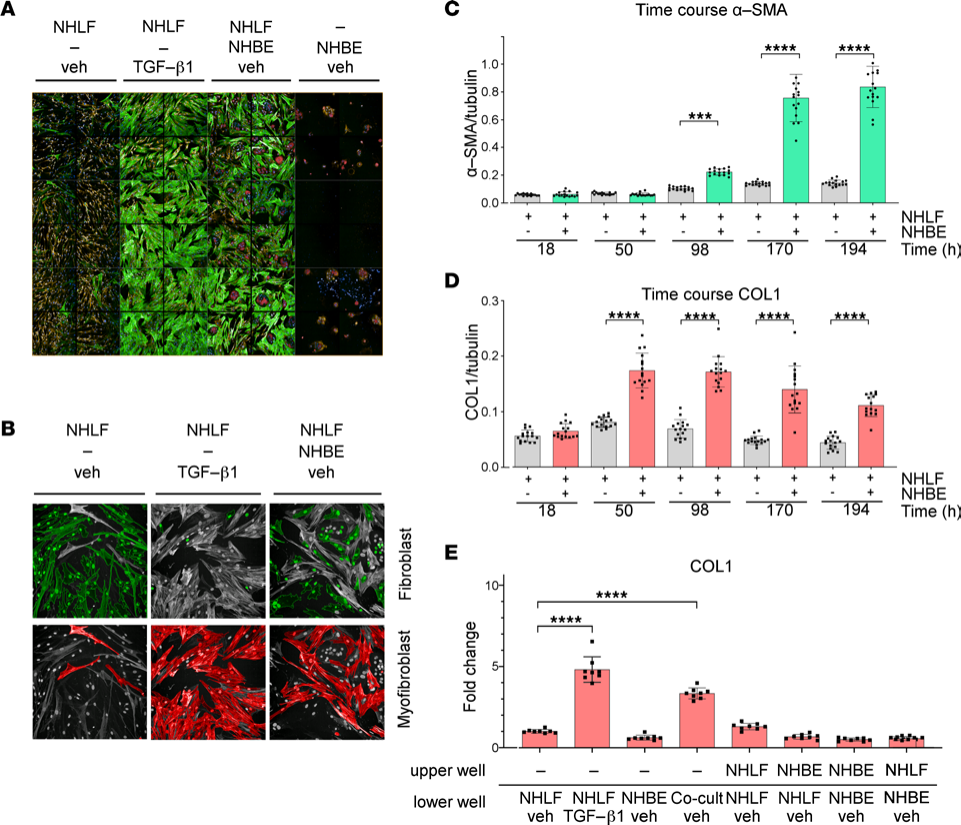
EC induce NHLF-to-myofibroblast transformation by cell-cell contact. (**A**) High-content confocal microscopy images of NHLF, NHLF stimulated with TGF-β1 (5 ng/mL), NHLF cocultured with NHBE, and NHBE cultured alone are shown. NHBE cells were pre-stained with CellTracker Deep Red. Nuclei were stained with DAPI, and α-SMA staining was performed using an anti–α-SMA antibody. (**B**) At 5 days after seeding cells were classified into EC, FB, and myofibroblast, respectively, by a trained classifier (see Methods for details). Classified FB and myofibroblasts are colored in green and red, respectively. Original magnification, ×20. (**C**) Time course of α-SMA and (**D**) COL1 accumulation. At 18, 50, 98, 170, and 194 hours after seeding, α-SMA and COL1 were quantified by MS/MS and plotted, normalized to tubulin, against time (hours). A 1-way ANOVA with Tukey’s multiple-comparison test was used. *n* = 16 for each condition. (**E**) COL1, normalized to tubulin, was detected by MS/MS after 98 hours in Transwell cell cultures of NHBE and NHLF seeded either together in the lower chamber or individually in the upper or lower chamber in the presence of 5 ng/mL TGF-β1 or vehicle as indicated. *n* = 8 for each condition. A 1-way ANOVA with Dunnett’s multiple-comparison test was used. Bars indicate mean ± SD. ***P ≤ 0.001, ****P ≤ 0.0001.

These observations prompted us to investigate potentially pathological interactions between EC and FB, and we cocultivated these 2 cell types in vitro. To this end, NHBE were prestained with CellTracker Deep Red and cocultured with NHLF in a 2-dimensional (2D) tissue culture plate. As controls, NHBE and NHLF were cultivated in monocultures in the absence of any supplements (veh), or monocultured NHLF were treated with TGF-β1, respectively, to induce myofibroblast transition. After 5 to 8 days the cells were fixed and stained with DAPI and an anti–α-SMA antibody for image analysis (Figure 2A).

Remarkably, in coculture, NHBE induced pronounced transition of NHLF to α-SMA–positive myofibroblasts. The effectiveness was comparable to NHLF that were stimulated with the known profibrotic mediator TGF-β1 in the absence of NHBE (Figure 2A). The effective induction of FB-to-myofibroblast transition in coculture was verified after cell segmentation into α-SMA–negative FB or α-SMA–positive myofibroblasts, using a trained classifier (Figure 2B).

When cocultivated with other epithelial cell types, NHLF also differentiated into cells with a myofibroblast-like phenotype, albeit with different effectiveness (Supplemental Figure 3, A and B). This was not, or only weakly, observed when NHLF were cocultured with NHLF, smooth muscle cells, or endothelial cells (Supplemental Figure 3C).

Surprisingly, a very low number of NHBE (i.e., 200 per well) was sufficient to induce a robust induction of α-SMA expression in the maximum number of tested NHLF (i.e., 4,000 per well; Supplemental Figure 3D). The effects were comparable when IPF patient–derived human lung fibroblasts instead of NHLF were used as effector cells (Supplemental Figure 3, A and C).

To complement our microscopy-based analysis results, we applied tandem mass spectrometry (MS/MS) protein analysis to accurately quantify cellular COL1A1 (herein abbreviated as COL1 for simplicity) and α-SMA protein levels from coculture lysates (19) at different time points after seeding. COL1 and α-SMA served as endpoints for ECM production and contractility, respectively. In NHLF/NHBE cocultures a significant increase in α-SMA protein was first detected after 98 hours, whereas a significant increase in COL1 protein was already observed 50 hours after seeding, suggesting that these markers may be controlled by different regulatory mechanisms (Figure 2, C and D).

To test whether the EC-induced FB-to-myofibroblast transition required direct cell-to-cell contact, we performed a Transwell experiment. In this experiment NHLF and NHBE were seeded either together in the lower chamber or individually in the upper or lower chamber. This allowed cocultivation of cells in the same chamber but without physical contact. Notably, increased COL1 accumulation after 98 hours was only observed when NHBE and NHLF were seeded in direct contact (Figure 2E).

To verify that secreted factors were not driving EC-induced activation of FB into myofibroblasts, conditioned monoculture supernatants from either NHBE or NHLF were added to FB, and cell responses were quantified using impedance technology. This methodology detects FB-to-myofibroblast transformation with high sensitivity and accuracy (19). The addition of supernatant of neither NHBE nor vehicle-treated NHLF led to any detectable impedance change (Supplemental Figure 4A). Interestingly, the addition of supernatant from NHLF/NHBE coculture to NHLF led to a transient increase in impedance with a peak after 4 hours (Supplemental Figure 4). This is indicative of a signaling event and suggested the presence of a coculture-specific factor, which was absent in supernatants of monocultured cells. Nevertheless, the addition of this secreted coculture factor was, in contrast to supernatant from TGF-β1–treated NHLF, insufficient to trigger an increase of α-SMA and COL1 in NHLF (Supplemental Figure 4, B and C).

In summary, the results indicate that EC, through cell-to-cell contact with FB, trigger transformation of FB into active myofibroblasts in cocultures.

### Cocultures of EC and FB induce pro-inflammatory and pro-fibrotic signaling pathways.

**Figure 3:**
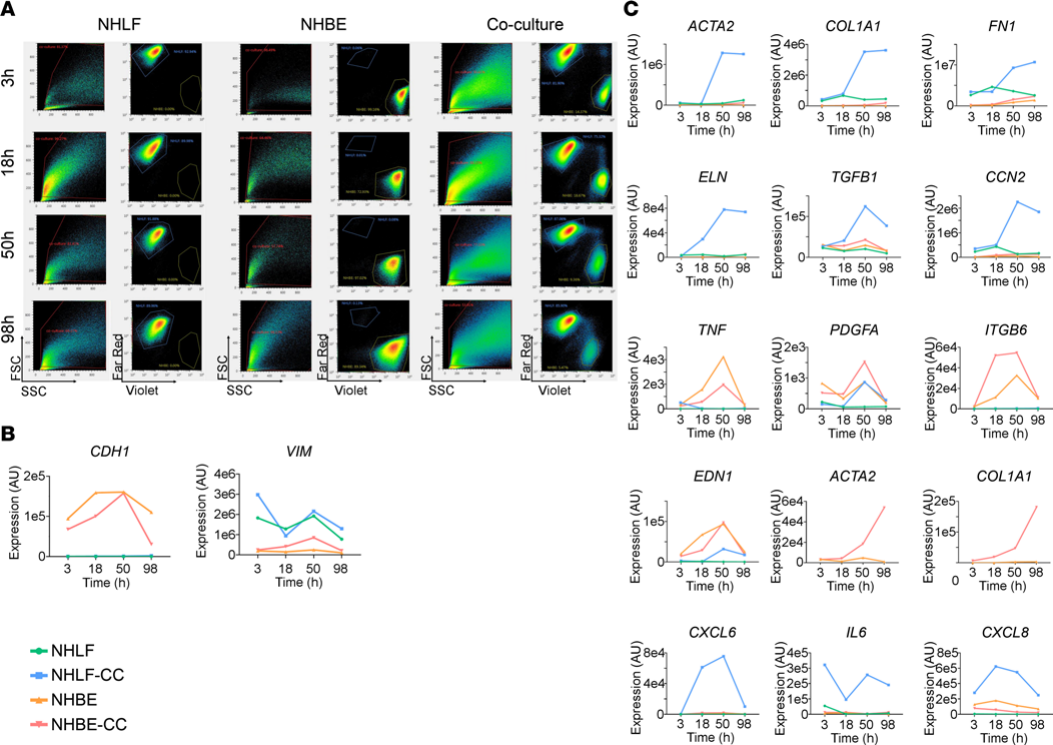
Pro-inflammatory and pro-fibrotic genes are induced in cocultured NHLF and NHBE. (**A**) For each condition, NHLF were prestained with CellTrace Far Red and the NHBE with CellTrace Violet; seeded, either separately or in combination as depicted, at *t* = 0 hours; and then FACS-sorted, followed by lysis at *t* = 3 hours, 18 hours, 50 hours, and 98 hours. Left-hand charts show scatterplots based on forward (FSC) and side scattering (SSC) profiles. Red line indicates gating threshold. Right-hand panels: Gated cells were sorted on CellTrace Far Red and CellTrace Violet staining intensities, respectively. (**B** and **C**) Selected genes are shown to address identity, inflammatory and profibrotic gene expression changes of the involved cell types over time. Expression level of *CDH1* and *VIM* (**B**) *ACTA2*, *COL1A1*, *FN1*, *ELN*, *TGFB1*, *CCN2*, *TNF*, *PDGFA*, *ITGB6*, *EDN1*, *CXCL6*, *IL6*, and *CXCL8* (**C**) in either monocultured NHBE and NHLF or cocultured NHBE (NHBE-CC) and NHLF (NHLF-CC). Relative expression levels, as determined by qRT-PCR and depicted as arbitrary units, are plotted versus time (hours). The experiment was performed once with *n* = 2–3 replicates per sample.

To follow cell type–specific temporal changes of gene expression in cocultured NHBE and NHLF, we used fluorescence-activated cell sorting (FACS) and quantitative real-time polymerase chain reaction (qRT-PCR) analysis. To this end, prelabeled NHBE (CellTrace Violet) and NHLF (CellTrace Far Red) were harvested at different time points from either monocultured or cocultured conditions and sorted by FACS. Both dyes were readily absorbed by the cells, stained the cells for the full duration of the experiment (i.e., 98 hours), and remained specific for each cell type in coculture conditions (Figure 3A). To study gene expression changes, we first used a limited set of literature-based fibrosis-associated and cell type marker genes (6, 18, 20).

The FACS-sorted NHBE expressed the epithelial marker gene cadherin 1 (*CDH1*) (Figure 3B), which was absent from NHLF populations. The FB marker vimentin (*VIM*) was predominant in FACS-sorted NHLF (Figure 3B), demonstrating that both prelabeled cell types were successfully separated after coculture.

NHLF, when cocultured with NHBE, showed a temporal expression increase of genes encoding ECM proteins and proteins associated with a myofibroblast phenotype, such as actin α2, smooth muscle (*ACTA2* coding for α-SMA); *COL1A1*; fibronectin 1 (*FN1*); and elastin (*ELN*), but also of *TGFB1* (coding for TGF-β1) and cellular communication network factor 2 (*CCN2*, coding for CTGF; Figure 3C). These genes showed similar kinetics, with a marked increase between *t* = 18 hours and *t* = 50 hours, which is in line with the fibrotic effector function of NHLF in this assay. NHBE in monoculture or in coculture expressed, in addition to *ITGB6*, a set of genes coding for secreted pro-inflammatory or pro-fibrotic proteins, tumor necrosis factor (*TNF*), platelet-derived growth factor subunit A (*PDGFA*), and endothelin 1 (*EDN1*). Expression of these genes transiently increased, reaching a peak at *t* = 50 hours, and subsequently decreased to baseline at *t* = 98 hours (Figure 3C). Interestingly, with time, expression of *ACTA2* and *COL1A1* was also detected in cocultured NHBE, but not monocultured NHBE (Figure 3C), suggesting epithelial-mesenchymal transition in cocultured conditions. Cocultured NHLF were the main contributing cell type to the expression of *TGFB1* and *CCN2* as well as to the cytokines *CXCL6*, *IL6*, and *CXCL8*. Remarkably, *CXCL6*, *CXCL8*, and *IL6* expression was elevated already at *t* = 3 hours in cocultured NHLF compared with monocultured NHLF. In summary, cell type–specific analysis of gene expression in NHLF/NHBE cocultures revealed induction of pro-inflammatory and pro-fibrotic gene expression.

### Early gene expression changes reveal NHBE-induced inflammatory responses in NHLF.

To learn more about the initiating stimulus in cocultures, we performed a comprehensive transcriptional characterization of the early events of the coculture. NHLF and NHBE cells were prestained (as above) and grown individually or as cocultures. Cells were collected at the time points *t* = 0 hours, 3 hours, and 18 hours, FACS-sorted to produce pure NHBE-derived and NHLF-derived cell population samples (Supplemental Figure 5); and analyzed by bulk RNA-Seq.

**Figure 4:**
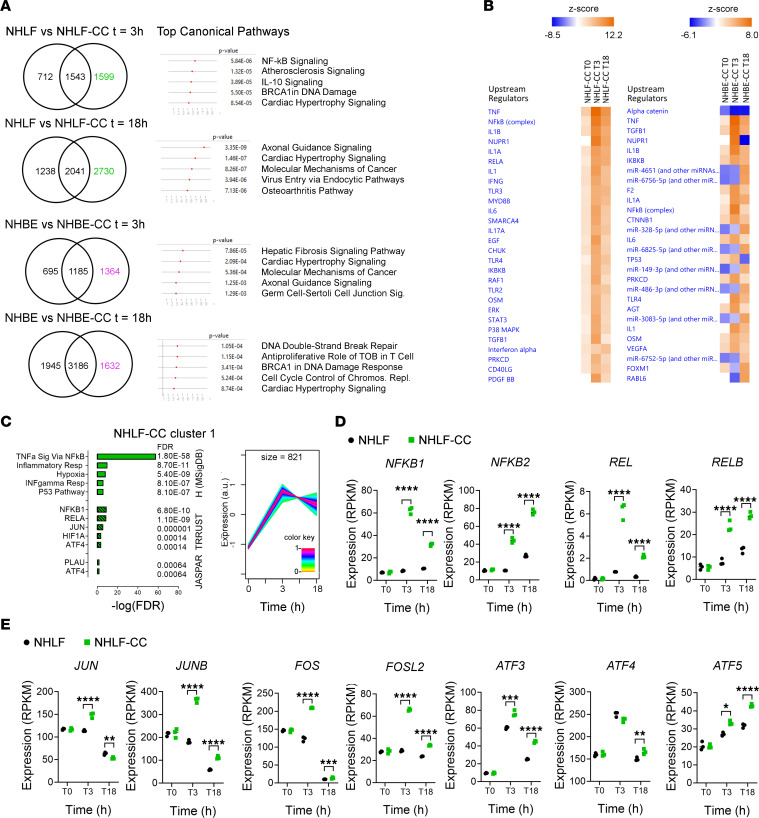
Early gene expression changes reveal coculture-induced NF-κB and AP-1 gene expression signature in NHLF and NHBE. (**A**) Venn diagrams displaying the number of overlapping differentially expressed genes (DEGs) per condition compared to *t* = 0 hours (FDR < 0.05 and |LinFC| > 1.5) and the top-ranked canonical pathways predicted for the coculture-specific genes, as identified by ingenuity pathway analysis (IPA) application. (**B**) Ingenuity upstream regulator analysis of the DEGs between cocultured and monocultured NHLF and NHBE (FDR < 0.05 and |LinFC| > 1.5) at *t* = 0 hours, 3 hours, and 18 hours, respectively, sorted according to the activation *z* score. (**C**) Expression kinetics and results of the GSOA of NHLF-CC cluster 1. The combined expression kinetics of the clustered genes is depicted as a *z* score (from –1 to 1) across the time points *t* = 0 hours (T0), 3 hours (T3), and 18 hours (T18), respectively. The expression pattern of each gene is associated with a cluster weight between 0 and 1 (according to its match with cluster dynamics), color-coded in the figure according to the inserted palette. Enriched gene sets are displayed as bars representing the –log_10_(FDR) with the corresponding FDR values. Gene expression, depicted in reads per kilobase of transcript, per million mapped reads (RPKM), of selected (**D**) NF-κB and (**E**) AP-1 TF family members, respectively, showing gene expression increases between conditions NHLF and NHLF-CC at the time points *t* = 0 hours, 3 hours, or 18 hours. DEGs in comparison with monocultured control were evaluated using edgeR and are depicted as *FDR ≤ 0.05, **FDR ≤ 0.01, ***FDR ≤ 0.001, and ****FDR ≤ 0.0001. The experiment was performed once with *n* = 3 replicates per sample.

First, we identified differentially expressed genes (DEGs) with an absolute linear fold-change (|LinFC|) > 1.5 and a false discovery rate (FDR) < 0.05 in the coculture and the respective monoculture at *t* = 3 hours and *t* = 18 hours, respectively. For all 4 conditions (i.e., NHLF, NHBE, NHLF-CC, and NHBE-CC), genes were included if expression was significantly different from expression at time *t* = 0 hours. Compared with the corresponding monoculture, 1,599 (*t* = 3 hours) and 2,730 (*t* = 18 hours) genes were specifically differentially regulated in the NHLF-CC and 1,364 (*t* = 3 hours) and 1,632 genes (*t* = 18 hours), respectively, in NHBE-CC (Figure 4A and Supplemental Table 1). Canonical pathway analysis, as implemented in the ingenuity pathway analysis (IPA) application, was performed on the DEGs that were specific for the cocultures to identify top-ranking canonical pathways (Figure 4A and Supplemental Table 2). IPA upstream regulator analysis on the coculture-specific DEGs revealed that already after 3 hours both NHLF-CC and NHBE-CC mounted inflammation- and TLR-mediated signaling, i.e., via TNF, cytokines of the IL-1 family, and IL-6, with the concurrent activation of the transcription factors NF-κB and STAT3 (Figure 4B and Supplemental Tables 3 and 4).

To identify coregulated gene clusters with similar temporal expression specifically during the early phases of coculture, noise-robust soft clustering based on the fuzzy c-means algorithm was performed on the gene expression time series data, excluding genes with low expression, i.e., max (count) transcripts per kilobase million < 1. A total of 6,289 DEGs in NHLF-CC and 6,295 DEGs in NHBE-CC were subjected to hierarchical clustering, and 7 was estimated as the most suitable number of clusters for each cell population. Hence, for both NHLF-CC and NHBE-CC, 7 clusters based on common gene expression kinetics were generated (Supplemental Figure 6). Next, a gene set overexpression analysis (GSOA) was performed to test whether certain biological functions or processes, or the consensus binding sites of transcription factors (TFs), were enriched in the genes associated with the coculture-specific time series clusters.

Querying the Hallmark (MSigDB) collection identified the gene set TNF-α signaling via NF-κB as highly enriched in NHLF-CC as well as NHBE-CC clusters that showed markedly increased gene expression between *t* = 0 hours and *t* = 3 hours (Figure 4C and Supplemental Figure 6, A, F, G, H, M and N). Querying of JASPAR (21) and TRRUST (22) collections, using the R package hypeR (23) with gene sets provided by Enrichr (24), revealed an overrepresentation of TF consensus binding sites and TF targets of the NF-κB as well as AP-1 family TFs in the coexpressed genes of those clusters that were characterized by the enrichment of the gene set TNF-α signaling via NF-κB (Supplemental Figure 6, A, G, H, and N).

We next examined whether the findings of the pathway analysis were also reflected in the gene expression of the individual NF-κB family members. Indeed, the expression of the NF-κB family members NF-κB subunit 1 (*NFKB1*) and subunit 2 (*NFKB2*); REL proto-oncogene, NF-κB subunit (*REL*); and RELB proto-oncogene, NF-κB subunit (*RELB*) showed coculture-specific upregulation at *t* = 3 hours and *t* = 18 hours (Figure 4D). Furthermore, in NHLF-CC, the expression of the AP-1 TF subunits Jun proto-oncogene (*JUN*), JunB proto-oncogene (*JUNB*), Fos proto-oncogene (*FOS*), and Fos like 2 (*FOSL2*) were upregulated at *t* = 3 hours versus *t* = 0 hours (Figure 4E) and in coculture versus monoculture. Activating transcription factor 3 (*ATF3*), 4 (*ATF4*), and 5 (*ATF5*) expression was increased at *t* = 3 hours versus *t* = 0 hours and increased in NHLF-CC versus NHLF at *t* = 3 hours and/or *t* = 18 hours (Figure 4E). Several AP-1 and NF-κB family subunits appeared upregulated at *t* = 3 hours in both mono- and cocultured NHBE, indicating that in NHBE expression changes of these genes are affected by culture rather than coculture (Supplemental Figure 7).

In summary, our expression analysis and GSOA of the early time points of the NHBE/NHLF coculture indicate that cocultivation triggers a strong acute inflammatory response, particularly in NHLF-CC, with increased expression and/or activation of the TFs NF-κB, AP-1, and STAT3.

### NF-κB signaling during the early phase of the coculture is essential for the fibrogenic activation of FB.

To address the translational relevance of this in vitro coculture system to investigate anti-fibrotic treatment modalities, we assessed the 2 IPF standard-of-care treatments, nintedanib and pirfenidone. To this end, compounds were added at the time of seeding of NHLF and NHBE cells, and changes in protein expression were quantified 98 hours later using MS/MS.

**Figure 5:**
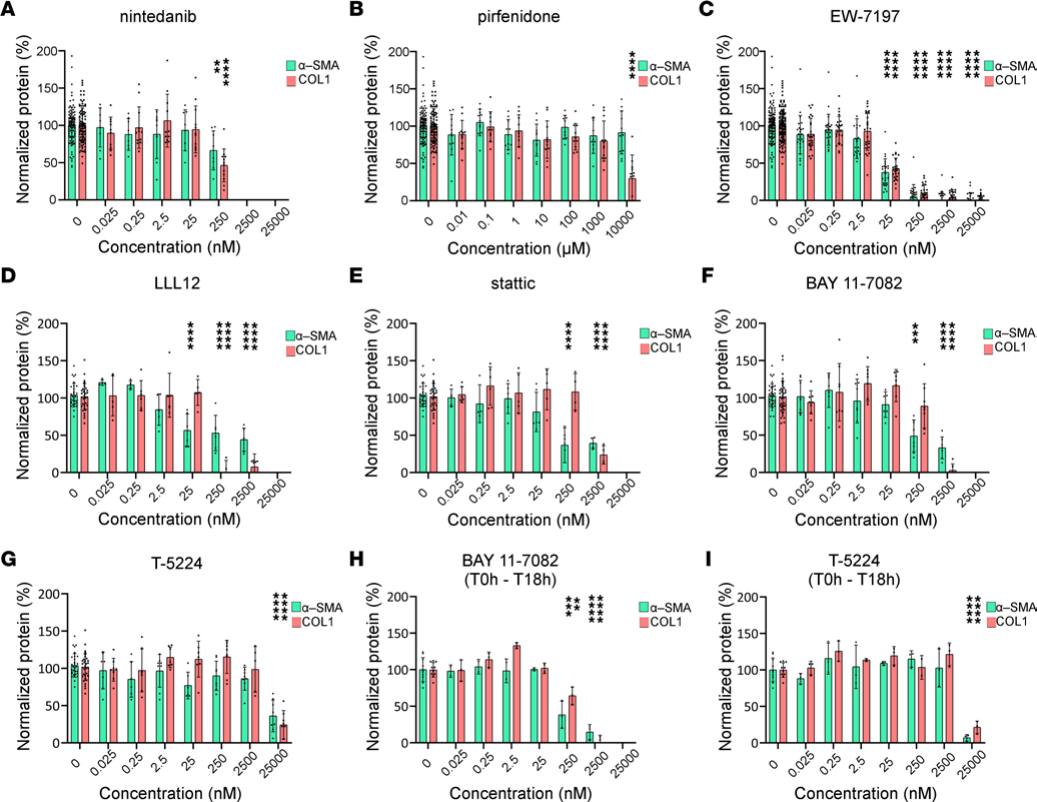
Pharmacological intervention attenuates α-SMA and COL1 accumulation in cocultures of NHBE and NHLF. NHLF and NHBE were seeded in full medium in the presence of (**A**) nintedanib, (**B**) pirfenidone, (**C**) EW-7197, (**D**) LLL12, (**E**) stattic, (**F** and **H**) BAY 11-7082, and (**G** and **I**) T-5224, at the indicated concentration range (0.025–25,000 nM), except for pirfenidone (0.01–10,000 μM). At *t* = 18 hours after seeding, cells were switched to starvation medium containing compounds at the indicated concentration (**A**–**G**) or solvent (**H** and **I**) for the remaining duration of the experiment. Lysis was performed 98 hours after seeding, and α-SMA and COL1 were quantified by MS/MS. The mean value of the normalized analyte level of the solvent control is given as 100%. Bars represent protein data normalized to tubulin (expressed as %) in relation to the solvent control and show mean ± SD; *n* = 8 (**A** and **D**–**G**), *n* = 11 (**B**), *n* = 26 (**C**), and *n* = 3 (**H** and **I**). Data for which cytotoxic effects are not excluded are omitted. A 2-way ANOVA with Tukey’s multiple-comparison test was used. *P* values of comparison to solvent control are depicted. **P ≤ 0.01, ***P ≤ 0.001, ****P ≤ 0.0001. In all wells, 0.25% DMSO was present.

Nintedanib showed a dose-dependent inhibition of both COL1 and α-SMA proteins, at concentrations for which no cytotoxicity was observed in a combined viability and toxicity assay on CHO cells (Figure 5, Supplemental Figure 8C, and Supplemental Table 5). Pirfenidone inhibited COL1 but not α-SMA accumulation, and this was only observed and statistically significant at the highest tested concentration (i.e., 10 mM; Figure 5B and Supplemental Table 5). Hence, both nintedanib and pirfenidone were able to inhibit COL1 production.

TGF-β1 is one of the best-described inducers of pro-fibrotic signaling, and its expression was upregulated in the coculture after 18 hours (Figure 3C). The known TGF-β receptor inhibitor EW-7197 (25) potently and concentration dependently inhibited coculture-induced COL1 and α-SMA protein accumulation (Figure 5C and Supplemental Table 5).

Cell type–dependent phosphorylation and activation of STAT3 has been attributed to IL-6 signaling via its receptors (26). The 2 inhibitors of IL-6/STAT3 signaling, LLL12 (27) and stattic (26), inhibited the accumulation of COL1 and α-SMA (Figure 5, D and E, and Supplemental Table 5) at concentrations that led to a reduction in the metabolic activity, but this was not associated with cytotoxicity (Supplemental Figure 8, F and G).

AP-1 and NF-κB signatures were identified as very early upregulated signaling pathways in the coculture model. BAY 11-7082 is a nonselective inhibitor of IκBα kinase IKK1 and IKK2, blocks the TNF-inducible phosphorylation of IκBα, and, thus, is expected to inhibit all forms of NF-κB (28), whereas T-5224 is a known inhibitor of protein c-FOS/AP-1 signaling (29). T-5224 and BAY 11-7082 effectively attenuated coculture-induced COL1 and α-SMA accumulation (Figure 5, F and G, and Supplemental Table 5) at concentrations previously shown to inhibit NF-κB and c-FOS/AP-1 in vitro (28, 29). The effects were not associated with cytotoxicity (Supplemental Figure 8). When compound exposure was restricted to the first 18 hours of coculture and was then removed, both BAY 11-7082 and T-5224 completely and concentration dependently abolished COL1 and α-SMA accumulation at *t* = 98 hours with a potency comparable to the standard protocol with continued exposure (Figure 5, H and I).

AP-1 and NF-κB have been found to enhance the expression of numerous genes involved in the inflammatory process (30). As assessed by Luminex assays, treatment with the NF-κB inhibitor BAY 11-7082 applied from *t* = 0 hours to *t* = 18 hours and then removed strongly inhibited the accumulation of IL-6, IL-8, and CXCL6 in the culture supernatant at *t* = 98 hours (Supplemental Figure 9B), whereas c-FOS/AP-1 inhibition with T-5224 did not influence their levels (Supplemental Figure 9C). TNF was below the limit of detection.

In summary, the results obtained with the pharmacological inhibitors provide supportive evidence for an active and causal contribution of TGF-β–, AP-1–, NF-κB–, and STAT3-mediated signal transduction with a critical involvement of c-FOS/AP-1 and NF-κB during the early phase of the coculture. Furthermore, NF-κB signaling during the early phase of the coculture was essential for the accumulation of the cytokines IL-6, IL-8, and CXCL6, whereas c-FOS was dispensable.

### Transcriptional expression profiles of in vitro–cocultured NHLF and NHBE resemble those of IPF-specific lung cell populations.

We further evaluated the fate of NHBE-CC and NHLF-CC during cocultivation and compared the genomic profiles of these cells at different time points with genomic profiles of cells that were isolated from patients with IPF.

**Figure 6:**
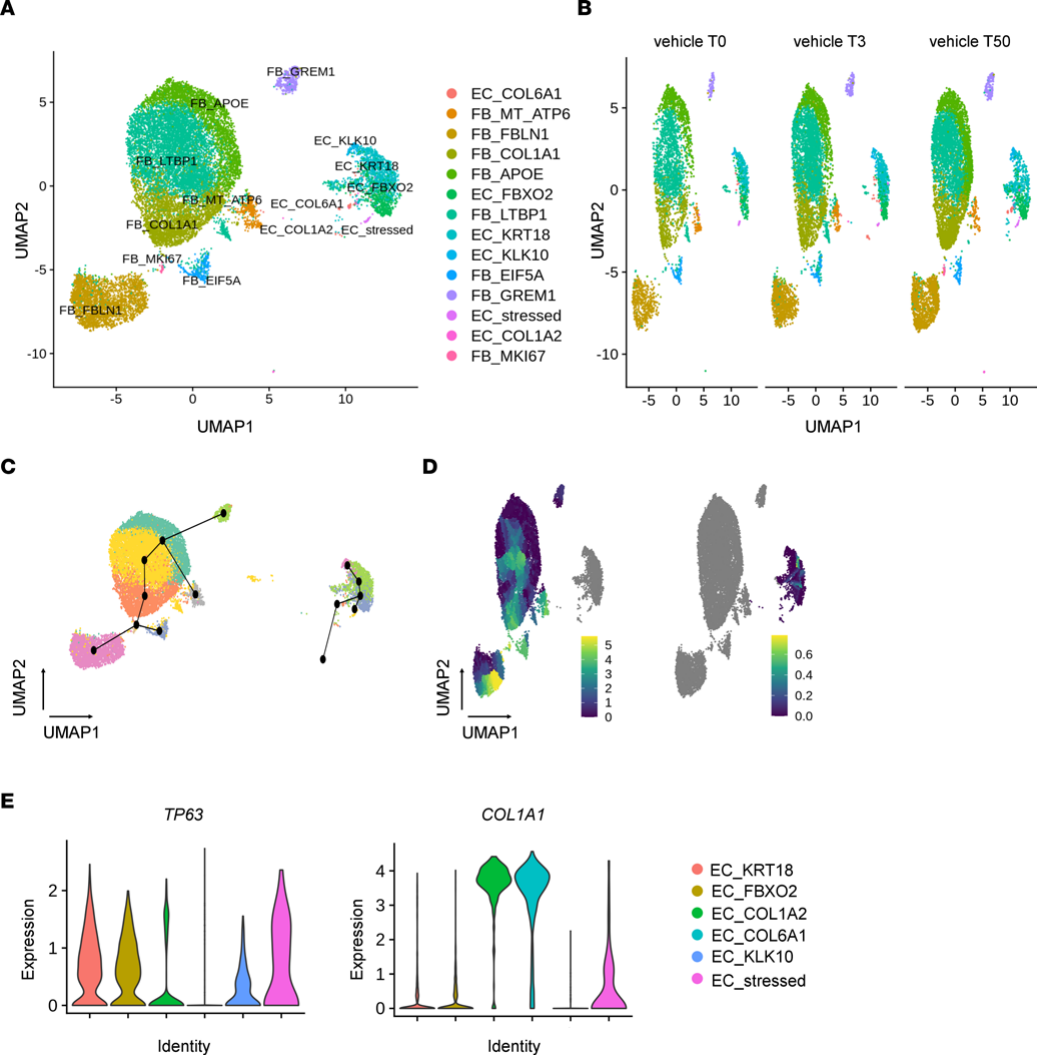
Single-cell landscape of NHBE and NHLF after 0 hours, 3 hours, and 50 hours of cocultivation. (**A**) Uniform manifold approximation and projection (UMAP) embedding of jointly analyzed single-cell transcriptomes from 18,676 cells from vehicle control. Cells were collected at 3 different time points (*t* = 0 hours, 3 hours, and 50 hours) in a single experiment. The 14 identified cell states were named after one of the most significant and cell state–specific marker genes as determined by the FindConservedMarkers function implemented in Seurat. (**B**) UMAP embedding, (**C**) Slingshot-based, and (**D**) Monocle 3–based pseudo-time trajectories calculated from UMAP embeddings of jointly analyzed 2,011 vehicle-treated EC, and 16,665 FB, including all time points (*t* = 0 hours, 3 hours, and 50 hours). (**E**) Violin plots displaying the expression level of *TP63*, and *COL1A1*, separated by substate. Normalized gene expression is depicted as log(counts+1).

To this end, droplet-based sc-RNA-Seq of NHBE-CC and NHLF-CC suspensions, collected at time points *t* = 0 hours, 3 hours, and 50 hours, was performed. After normalization and integration of the data sets, we performed graph-based clustering toward identifying cell states across the different experimental variables. This analysis recovered 14 cell substate clusters across NHBE-CC and NHLF-CC lineage cells (Figure 6A). Each of the 6 EC and the 8 FB cell substates was named after one of the most significantly associated genes for each state (see Methods for details).

Annotation of the UMAP embedding of the vehicle-treated cells according to the time of collection showed that cells of all substates were collected at each time point (Figure 6B). To capture any underlying dynamic processes or to identify trajectories between cell substates, we applied trajectory inference methods, also called pseudo-time analyses (31). Pseudo-time analysis, performed using the Slingshot (32) package, generated branched trajectories connecting the clusters of the FB and EC populations, respectively (Figure 6C). For the EC population, Slingshot created a branched trajectory along which cells of the EC_COL6A1 and EC_COL1A2 substates separated from the main EC population trajectory at EC_FBXO2 (Figure 6C). These 2 distinct branches segregated the 6 distinct EC substates based on the presence or absence of *COL1A1* and tumor protein p63 (*TP63*) expression (Figure 6E). Pseudo-time analysis using Monocle 3 (33) allowing for more refined resolution of cell states (clusters) revealed temporal gene expression changes within the substates of both populations, i.e., FB and EC (Figure 6D).

**Figure 7:**
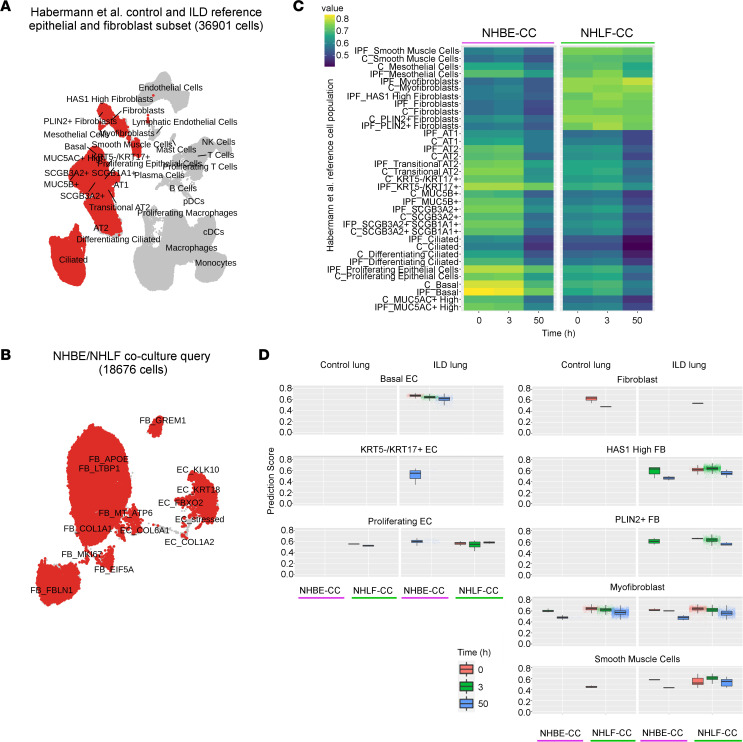
The genomic expression signature of NHBE and NHLF in the coculture shows similarity to cell populations isolated from IPF patient lungs. (**A**) UMAP embedding of the Habermann et al. data set (5). Lung mesenchymal and epithelial cell types highlighted in red were carried forward to generate a reference data set comprising 36,901 epithelial and mesenchymal cells representing control and interstitial lung disease (ILD) subsets. (**B**) All cells corresponding to the vehicle group time points *t* = 0 hours, 3 hours, and 50 hours, highlighted in red, were included from the coculture system and served as the query data to probe the reference data set. (**C**) Pearson’s correlations of vehicle-treated NHBE-CC and NHLF-CC query populations with human IPF (IPF) and control (C) reference populations. (**D**) Iterative Spearman’s correlations at single-cell level between marker genes shared by both query and Habermann et al. reference population, and depicted as overall SingleR correlation prediction scores of vehicle group cell types in human lung.

To evaluate the consequences of the gene expression dynamics, we determined the similarity of the coculture system with a human IPF patient lung cell atlas (5). Of the published reference data set, we used the IPF patient–derived and control lung mesenchymal and epithelial types to generate a reference atlas with reduced dimensionality, keeping the author’s original cell type labeling (Figure 7, A and B).

To compare our in vitro culture with human lung data, we calculated Pearson’s correlations of the averaged expression of shared variably expressed genes, and used SingleR (34) to calculate, at the single-cell level, Spearman’s correlations of the expressed marker genes that were shared between reference and query cells. At *t* = 0 hours, some NHLF-CC query cells paired up with “Fibroblasts,” but across all time points the FB population matched most closely the reference populations “HAS1 High FB,” “PLIN2+ FB,” “Myofibroblast,” and “Smooth Muscle Cells” (Figure 7, C and D, and Supplemental Table 6). If only the human reference population with the highest similarity to the respective query cell was considered, the NHLF showed the highest similarity to “Myofibroblast” at baseline (94%) and at *t* = 50 hours (98%; Table 1). At the intermediate time point, i.e., 3 hours after seeding, 50% and 21% of the query FB population showed the highest similarity to the “HAS1 High FB” and the “PLIN2+ FB,” respectively (Table 1). At *t* = 3 hours, the cells resembling the “HAS1 High FB” were composed mainly of the FB_LTBP1 (49%) state, which also contributed 37% of the cells to the pool showing similarity to “PLIN2+ FB.” Since the classical myofibroblast marker genes showed upregulation only from *t* = 50 hours (Figure 3), the unbiased correlation revealed that the NHLF displayed gene expression patterns of myofibroblasts from the outset and thus may have adopted a “pre-myofibroblast” state during cultivation. Furthermore, our data suggest that the “HAS1 High FB” and “PLIN2+ FB” populations may represent intermediate stages in the course of transforming into fully differentiated ECM-synthesizing myofibroblasts. For the joint EC query population, the best Pearson’s correlations were obtained for “Basal EC,” “KRT5-/KRT17+ EC,” and “Proliferating EC” from patients with IPF (Figure 7C and Supplemental Table 6). SingleR revealed similarity of the query EC population with the “Basal EC” population from the start of the culture (Figure 7D). At *t* = 50 hours of cultivation, fractions of the EC query population (i.e., 8%, 6%, and 4%), adopted states most closely resembling the “Proliferating EC,” “Myofibroblast,” and “KRT5-/KRT17+ EC” populations from patients, respectively (Table 1). The pools resembling “Proliferating EC” were mainly composed of EC_KRT18 (69%) and EC_FBXO2 (28%) and “KRT5-/KRT17+ EC” were mainly composed of EC_COL1A2 (25%), EC_COL6A1 (15%), EC_FBXO2 (25%), and EC_KRT18 (30%). All correlations for the query at the level of the substates are provided in Supplemental Tables 7–9.

In summary, our analysis revealed a pre-myofibroblast profile for the NHLF at the beginning of culture. Subsequently, the majority of NHLF adopted “HAS1 High FB” and “PLIN2+ FB” identity before their complete conversion into myofibroblasts. The NHBE consisted largely of cells with basal cell signature, and the majority retained basal cell characteristics, with fractions of them assuming the character of “Proliferating EC,” “Myofibroblast,” and “KRT5-/KRT17+ EC.” Therefore, transcriptional profiles of in vitro–cocultured NHLF and NHBE resemble cell populations that are also enriched in the lungs of patients with ILD.

### NF-κB contributes to early molecular changes in cocultured FB and EC populations.

We next investigated whether NF-κB or AP-1 was involved in changes to the EC — or FB — populations, i.e., whether inhibition of IKK/NF-κB and c-FOS/AP-1 with BAY 11-7082 and T-5224, respectively, had potential to modulate EC or FB phenotypes.

**Figure 8:**
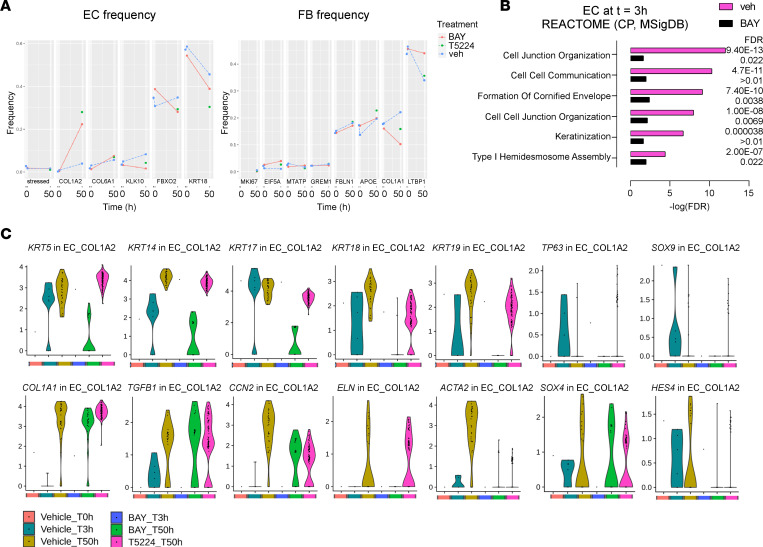
Inhibition of c-FOS/AP-1 and IKK/NF-κB affects cocultured epithelial cell population frequencies and marker gene expression at subpopulation resolution. (**A**) Effect of treatment — i.e., 2.5 μM BAY 11-7082 (BAY), 25 μM T-5224 (T5224), and vehicle (veh) — on epithelial lineage cell frequencies (*y* axis) separated by EC substate and plotted for each time point (*t* = 0 hours, 3 hours, and 50 hours; *x* axis). (**B**) Effect of the NF-κB inhibitor BAY 11-7082 (2.5 μM) applied from *t* = 0 hours, on enriched canonical pathways (CP, Reactome) in the EC DEGs at time *t* = 3 hours displayed as bars representing the –log_10_(FDR). (**C**) Violin plots displaying the expression level of *KRT5*, *KRT14*, *KRT17*, *KRT18*, *KRT19*, *TP63*, *SOX9*, *COL1A1*, *TGFB1*, *CCN2*, *ELN*, *ACTA2*, *SOX4*, and *HES4*, separated by sample and at the level of the subclustered cell state identifier EC_COL1A2. Cells were either untreated (vehicle) or treated (i.e., 2.5 μM BAY 11-7082, 25 μM T-5224) and collected in a single experiment at the time points (*t* = 0 hours, 3 hours, and 50 hours). Normalized gene expression is depicted as log(counts+1) for a nonstatistical overview of gene-of-interest expression in the data set.

As determined by MAST (35), both the culture time of 50 hours and the 2 pharmacological interventions changed the relative frequencies of the EC and FB populations (Figure 8A). In the EC population, both treatments markedly decreased the dominant basal EC substates characterized by low or absent *COL1A1* and high *TP63* expression (i.e., EC_KRT18, EC_FBXO2, and EC_KLK10; Figure 8A) and, consequently, increased the proportion of the *COL1A1*-expressing transitional substates (i.e., EC_COL1A2 and EC_COL6A2). Among the FB substates, both treatments reduced the proportion of FB_COL1A1, which is characterized by elevated expression of *ACTA2*, *CCN2*, and *IL6*. Furthermore, BAY 11-7082 and T-5224 blunted the expression increase of *ACTA2*, *CCN2*, and *IL6* within the FB_COL1A1 substate (Supplemental Figure 10A).

GSOA for the DEGs of cellular substates did not provide significant insights, likely due to the small population sizes. GSOA on the total EC population at *t* = 3 hours revealed overexpression of functions indicative of development, cell junction organization, cell-cell contact, adhesion, differentiation, cornification, and keratinization (Figure 8B and Supplemental Figure 11). The addition of BAY 11-7082 suppressed these functions, indicating that these biological processes are largely controlled by NF-κB signaling. Twenty-four of the 100 most significantly affected EC genes by each treatment (FDR < 0.01) were affected by both BAY 11-7082 and T-5224 (Supplemental Figure 10B).

The 2 *COL1A1*-expressing EC substates (EC_COL1A2 and EC_COL6A2) showed a dynamic and pronounced expression increase of *KRT5*, *KRT14*, *KRT17*, *KRT18*, and *KRT19*, indicative of ongoing differentiation, keratinization, and cornification. Furthermore, they showed increased expression of genes linked to development (*SOX9*), senescence (*SOX4*), Notch signaling (*HES4*), and epithelial-mesenchymal transition (i.e., *COL1A1*, *TGFB1*, *CCN2*, *ELN*, *ACTA2*, Figure 8C). In cluster EC_COL1A2, treatment with BAY 11-7082 prevented the increase of all detected keratins, whereas T-5224 blunted the expression increase of *KRT8*, *KRT17*, *KRT18*, and *KRT19* (Figure 8C). The dominant *TP63*-expressing basal EC substates (e.g., EC_KRT18) exhibited relatively stable keratin expression (Supplemental Figure 10C), and keratin expression was not markedly affected by the treatments. Hence, inhibition of IKK/NF-κB and c-FOS/AP-1 modulated FB and EC phenotypes, as judged by their effect on population composition, biological pathway activities, and keratin profiles. The culture and treatment effects were restricted to certain cell states and were, thus, context dependent.

### IPF patient lung–derived FB populations express the AP-1 and NF-κB signatures observed in the early stages of NHLF-CC and NHBE-CC.

**Figure 9:**
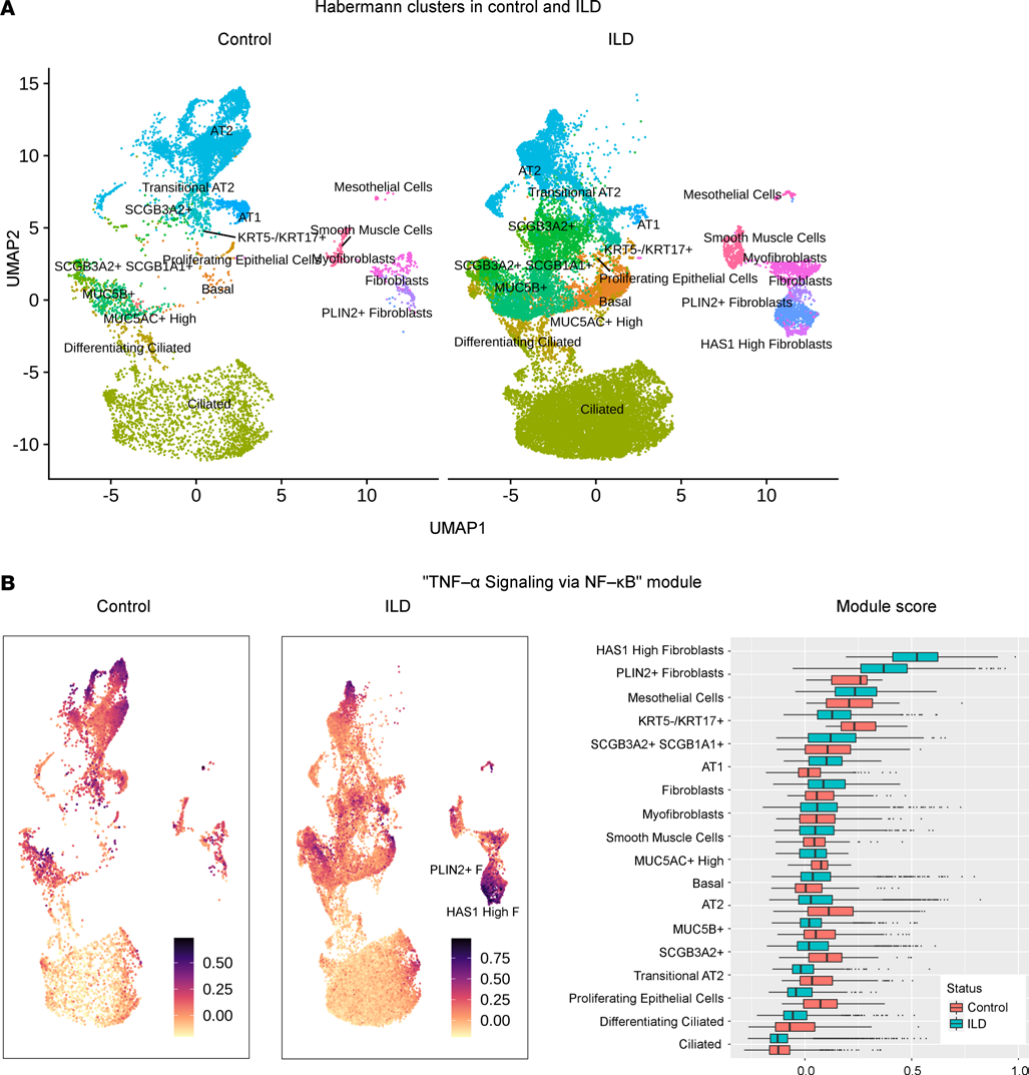
Expression of the NF-κB gene module in cell populations isolated from IPF patient lungs. (**A**) UMAP embedding of the Habermann et al. reference subset (5) comprising 36,901 epithelial and mesenchymal cells divided into control and ILD subsets. (**B**) Results of the module score analysis querying the Habermann reference human IPF patient lung cell atlas with the gene expression module TNF-α Signaling via NF-κB. The UMAP space of control and ILD cells is overlaid with the obtained module scores for each individual cell. Box plots display module scores (*x* axis) obtained for the respective reference cell populations (*y* axis) in control (red boxes) and ILD patient–derived (turquoise boxes) cells.

To assess whether AP-1 and NF-κB signatures that we identified in our coculture model were also expressed in patients with IPF, a module score analysis (36) was performed on the reference human IPF patient lung cell atlas (5). In a first step, the reference data set was partitioned into control lungs versus ILD (Figure 9A). We then assembled modules of AP-1– and NF-κB–associated genes that showed similar expression kinetics and increased expression after 3 hours in coculture. The AP-1 module consisted of the genes *JUN*, *JUNB*, *FOS*, *FOSL2*, *ATF3*, *ATF4*, and *ATF5*, and the NF-κB module comprised *NFKB1*, *NFKB2*, *REL*, *RELA*, and *RELB*. In addition, we examined a TNF module consisting of 84 coculture genes overlapping with the Hallmark gene set TNF-α signaling via NF-κB identified as enriched in the NHLF-CC after 3 hours (Figure 4C). Expression module analysis for all 3 tested gene sets showed the highest expression scores for the “HAS1 High FB,” as well as for the “PLIN2+ FB,” which were both more abundant in ILD lungs compared with control lungs (Figure 9B and Supplemental Figure 12).

### Interactome-based analysis identifies potential ligand-receptor binding pairs specific to EC-FB interactions.

**Figure 10:**
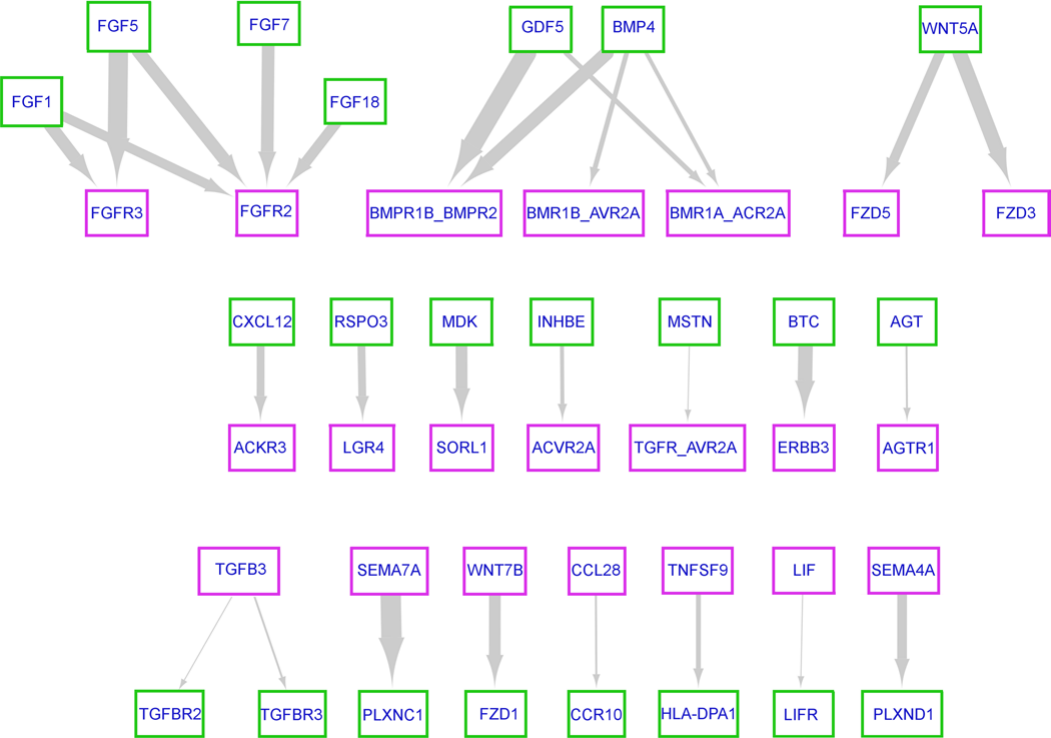
Predicted ligand-receptor pairs between FB and EC. Ligand-receptor pairs between FB (green box) and EC (pink box) were extracted from the sc-RNA-Seq data of the vehicle-treated cells at *t* = 0 hours using CellPhoneDB and visualized with Cytoscape. The arrowhead points to the receptor-expressing cell type. Arrow thickness correlates with the magnitude of the product of mean ligand expression and mean receptor expression for each cell type interaction.

Our results obtained thus far indicated that cell-to-cell contact between EC and FB was required. To identify potential ligand-receptor binding pairs involved in the activation of NHLF, we performed interactome analysis of the sc-RNA-Seq data of the vehicle-treated cells at *t* = 0 hours using CellPhoneDB (37). To specifically identify EC-FB pairings, the data set was cleared of those combinations that also occurred between EC-EC and FB-FB. Interactions between cells and matrix (integrins) were also removed since the pairing process takes place prior to significant ECM deposition. The generated interaction networks for FB and EC ligand-receptor pairs were visualized with Cytoscape (38) (Figure 10). Our analysis implied growth factor-driven signaling through fibroblast growth factor (FGF), bone morphogenic protein (BMP), activin, and receptor tyrosine protein kinase erbB-3 (ERBB3) receptors, respectively, as central mechanisms by which FB can interact with EC, as well as the frizzled class of receptors and the Leucine-rich repeat-containing G protein–coupled receptor 4 (LGR4) co-receptor for wingless (Wnt) signaling proteins. Semaphorin-7A (*SEMA7A*) and *SEMA4A*, in addition to TNF receptor superfamily member 9 (*TNFSF9*), are plasma membrane localized and could, hence, require cell-cell contact to engage their receptors (Figure 10). In addition to the *SEMA7A*>Plexin-C1 (*PLXNC1*) and *SEMA4A*>*PLXND1* pairs that were identified at *t* = 0 hours, *SEMA3A*, *SEMA3B*, and *SEMA4B* showed increased and NHBE-CC–specific expression at *t* = 3 hours and could interact with their corresponding plasma membrane–localized plexin receptors, or neuropilin co-receptors (i.e., *NRP2*) expressed on NHLF-CC (Supplemental Table 1). Therefore, EC-driven effects on FB may potentially involve, in addition to TGF-β–, Wnt-, and cytokine-mediated responses, specific semaphorin-mediated signaling via plexin receptors.

## Discussion

Dysregulated epithelial-mesenchymal interactions have been proposed as a disease-causing mechanism of IPF (3). However, there has been limited progress in developing an integrated understanding of the origin of the disease, as well as of central mechanisms driving pathological epithelial remodeling and ECM expansion in patients with lung fibrosis. We developed an in vitro coculture model using primary NHBE and NHLF that allowed us to investigate the dynamic molecular interactions between both cell types. Surprisingly, over a period of several days, coculturing both cell types resulted in the effective differentiation of NHLF into fibrotic mesenchymal effector cells, whereas NHBE initiated functions related to development, cell-cell contact, differentiation, keratinization, and eventually EMT. Notably, only epithelial cell types effectively triggered the differentiation of cocultured FB into α-SMA–positive myofibroblasts in our high-content assay, and specifically lung NHBE were the most effective in this assay.

Remarkably, the fibrotic process proceeded autonomously and without addition of external pro-fibrotic stimuli, e.g., TGF-β1, which is consistent with published data using similar conditions (11). However, when EC were used in 3D cocultures, they seemed to dampen and not promote the activation of cocultured FB (10, 12, 16). We also did not observe an increase in COL1/α-SMA when EC-FB interaction was studied in EC culture medium, but the decreased tubulin content suggested that NHLF did not tolerate these culture conditions well (data not shown). Hence, specific culture conditions can influence the outcome of epithelial-mesenchymal interactions, which is in line with previous reports (12–14, 16).

The fact that both cell types must be seeded in the same compartment suggests direct cell-cell contact as part of the driving mechanism. In the healthy lung, direct cell-to-cell contact between airway EC and FB is normally prevented by the basement membrane. However, in IPF, the extensive breakdown of lung architecture in areas of active disease may allow the establishment of uncontrolled contacts between the EC and FB, making it plausible that these 2 cell types directly influence each other and drive disease progression (5, 15, 39). Our interactome-based analysis has identified several potential ligand-receptor pairs that may mediate specific cell-to-cell signaling between EC and FB, and these will be evaluated in future studies. Semaphorin signaling, as one of the possible contributors, has not been thoroughly explored in the context of lung fibrosis and may provide a novel drug target. Semaphorins play a critical role in angiogenesis but have also been reported to modulate tumor microenvironment and to influence the biology of cancer-associated FB (40).

As revealed by our sc-RNA-Seq study, the commercially available NHBE preparations that were used in the coculture were substantially enriched for cells with a basal EC signature already at the time of seeding. Since the inflammatory and the subsequent fibrogenic response of the FB was initiated with almost immediate effect, it can be speculated that the observed effects were indeed promoted by EC with a basal cell signature. This would be in line with very recent literature demonstrating the profibrotic propensity of airway-derived basal cells (41). By applying 2 independent correlation analyses, we found that fractions of the seeded EC population adopted phenotypes resembling the “KRT5-/KRT17+ EC” and “Proliferating EC” populations, as identified in the reference IPF patient lung data set (5). However, the cells with the gene expression profile resembling the “KRT5-/KRT17+ EC” cells appear to arise only over a 50-hour cultivation period by differentiation (possibly by undergoing EMT), presumably from the predominant basal cell states EC_FBXO2 and EC_KRT18 and, possibly, by expansion of the EC_COL1A2 and EC_COL6A1 states. Thus, “KRT5-/KRT17+ EC” are unlikely to drive the fibrogenic response of the FB, and, hence, their role remains to be determined.

IPF is characterized by a high temporal and spatial heterogeneity (4–7, 9, 42, 43). Lack of synchrony complicates the drawing of conclusions on the ontology of the disease. In addition, the mechanisms driving IPF progression remain incompletely defined. In contrast, the synchronized progression of the cellular responses in our coculture model allows a temporal resolution of the underlying processes. The phenotypic shift of cocultured NHLF toward a myofibroblast phenotype was preceded by processes that showed inflammatory characteristics and involved the early activation of the “rapid-acting” transcriptional regulators STAT3, NF-κB, and AP-1 as revealed by our GSOA and differential expression analysis. Our correlation-based analyses revealed the highest similarities of the queried FB population at the 3-hour time point to fibrotic mesenchymal populations such as the “HAS1 High FB” and “PLIN2+ FB” of the reference atlas. Furthermore, our module score analysis using AP-1, NF-κB, and “TNF-α signaling via NF-κB” gene signatures achieved the highest expression scores for the “HAS1 High FB,” as well as for the “PLIN2+ FB,” of the IPF lung reference atlas. Thus, the signaling networks in cocultured NHLF early after the onset of culture and certain FB populations in patient lungs appear to be very similar, and both are dominated by AP-1 and NF-κB activity. Building on the temporal resolution of our model, it could be hypothesized that in patients the “HAS1 High FB” and the “PLIN2+ FB” cell populations may represent transition states toward terminally differentiated ECM-generating myofibroblasts.

The expression of *JUN* (coding for c-JUN) was increased in many human fibrotic diseases (44), and strong expression of *FOSL2* (encoding FRA-2) was observed in human samples of pulmonary fibrosis associated with vascular remodeling (45). Furthermore, in lung biopsy sections from patients with IPF, c-JUN and c-FOS were detected in a subset of FB in fibrotic areas (46). The systemic induction of c-Jun in mice leads to the development of multiorgan fibrosis with strong lung involvement (44), whereas ectopic expression of *FOSL2* results in lung fibrosis, associated with vascular remodeling of the pulmonary artery (45). Thus, activation of both c-FOS/AP-1 and c-JUN/AP-1 causes pulmonary fibrosis. Applying the c-FOS/AP-1 inhibitor T-5224 during the inflammatory phase (from *t* = 0–18 hours) of our model showed that c-FOS/AP-1 also played an essential and early role in the profibrotic differentiation of cocultured NHLF in myofibroblasts. In addition, inhibition of IKK/NF-κB by BAY 11-7082, during the first 18 hours of coculture, completely suppressed α-SMA and COL1 production in cocultured NHLF. Thus, in our model, the activation of the transcriptional regulators NF-κB and AP-1 occurred very early and was causative of the phenotypic shift of NHLF toward a myofibroblast phenotype. This is in line with recently published findings, which revealed that TGF-β receptor signaling and STAT3 signaling pathways are downstream of AP-1 TF c-JUN (46).

Genomic analysis of sorted NHBE at different time points of coculture identified Hallmark AP-1 and NF-κB activity. Pharmacological intervention using c-FOS/AP-1 and IKK/NF-κB inhibitors, T-5224 and BAY 11-7082, respectively, modulated the phenotypes of specific epithelial clusters, as judged by their effects on keratin profiles, cell state frequency, and biological function. Particularly pronounced in the *COL1A1*-expressing EC states (e.g., EC_COL1A2), the effects of IKK/NF-κB inhibition were observed already at *t* = 3 hours, suggesting direct and context-dependent transcriptional control of NF-κB on epithelial keratin expression, as well as functions associated with, e.g., keratinization, differentiation, and cell-cell interaction.

The cells in the described coculture lung fibrosis assay system showed aspects of phenotype changes and genomic Hallmark signatures that were found in cells from IPF patient lungs. However, the known limitations of 2D culture conditions, including the high stiffness of the culture vessel surface that might artificially influence the cell behavior, still apply. Furthermore, although the IPF patient–derived fibroblasts were examined without detecting major differences compared to NHLF, the same might not be true for IPF-derived lung EC. In particular, patient-derived basal EC should be considered in future studies, because they were not investigated here because of the lack of availability.

In summary, we described here the development and thorough characterization of an in vitro lung fibrosis model and demonstrated that it i) works autonomously, i.e., no added pro-fibrotic stimulants such as TGF-β are required; ii) depends on direct EC-FB contact; iii) develops into a fibrotic condition progressively and synchronously; and iv) leads to dynamic phenotype changes of EC and FB that are very similar to cells associated with progressive fibrogenic changes in patients with IPF. Furthermore, using this model, we identified NF-κB as a critical component in both the culture-induced phenotypic changes in EC and the differentiation of FB to myofibroblasts. Inhibition of NF-κB may have potential to suppress both the fibrotic foci and the appearance of aberrant EC in patients with IPF.

## Methods

A detailed description of all methods and materials used in this study can be found in the Supplemental Methods section.

### Coculture and protein quantification assay

#### NHLF/NHBE coculture.

NHLF were resuspended in FGM-2 growth medium (Lonza), mixed with an equal volume of NHBE in BEGM growth medium (Lonza), and seeded into a 96-well, flat-bottom culture plate (Corning) at a density of 20,000 and 1,600 cells per well, respectively. Compound dilution series were added, and the coculture was incubated for 18 hours at 37°C/5 % CO_2_. The medium was then replaced with Fibroblast Growth Basal Medium (Lonza) containing 0.1 % fatty acid–free BSA and the compounds at the indicated concentrations. The medium was supplemented with 100 U/mL penicillin, 100 μg/mL streptomycin, for the entire duration of the culture. If not indicated otherwise, the coculture was incubated at 37°C/5 % CO_2_ for 96 hours.

#### Lysis of cells for MS/MS analysis.

The cell culture medium was removed, and cells were lysed on ice. Samples were prepared for MS/MS, and surrogate tryptic peptides were chosen for detection of COL1 (COL1A1), α-SMA (ACTA2), and tubulin (TBA1A1) as described (19). Peak areas for COL1A1 and ACTA2 were normalized by dividing by TBA1A1 peak area.

### Gene expression analysis by bulk RNA-Seq

#### Library preparation, sequencing, and data processing.

Cells were isolated by FACS and lysed in RL Lysis Buffer from Single Cell RNA Purification Kit (Norgen Biotek Corp.). Total RNA was isolated using Single Cell RNA Purification Kit (Norgen Biotek Corp.) including a DNase treatment, according to the manufacturer’s instructions. RNA-Seq libraries of poly(A)-selected RNA were isolated from 50 ng of total RNA using NuGen (Tecan) Universal Plus mRNA-Seq Library Preparation Kit with NuQuant according to the manufacturer’s recommendations. For the sequencing runs, the samples were randomized across 2 flow cells and sequenced using NextSeq 500/550 High Output Kit v2.5 with 75 cycles (Illumina). Sequenced reads were aligned with STAR (v2.5.4b) to the human reference genome (GRCh38), and genewise alignments were quantified with featureCounts using Ensembl gene annotations. See Supplemental Methods for further information. All samples passed quality control. DEGs were evaluated using edgeR (47), where genes with an FDR < 0.05 and a LinFC > 1.5 were considered significant.

### Sc-RNA-Seq

#### Library preparation and sequencing.

Cells were resuspended in ice-cold FACS buffer (autoMACS Rinsing Solution, Miltenyi Biotec) supplemented with 0.5% fatty acid–free BSA (Calbiochem) to approximately 1 × 10^6^ cells/mL. At time point 0 hours, cells were combined directly in ice-cold FACS buffer. Average viability of all samples taken into single-cell sequencing was 92.5% (88.4%–94.8%). Cell suspensions were immediately loaded on a Chromium Next GEM Chip K (10x Genomics) using the Chromium Next GEM Single Cell 5′ Kit v.2 reagents according to the manufacturer’s recommendations. Final libraries were pooled and sequenced to an average depth of more than 42,000 reads per cell on an Illumina NovaSeq system by GENEWIZ.

#### Sc-RNA-Seq preprocessing.

Processing of raw data, filtering, normalization, and graph-based clustering are described in detail in the Supplemental Methods. For labeling of clusters, we used a Wilcoxon rank-sum test implemented in Seurat’s FindConservedMarkers function to determine cluster-specific marker genes independent of time point or treatment.

#### Sc-RNA-Seq analysis.

Augur (48) was used to predict culture time effect for each cell cluster. Pairwise differential expression across time points or treatments was performed using MAST (35) implemented in Seurat after merging all EC into 1 group. Expression testing was performed on the log-normalized counts. Results were filtered based on a Bonferroni-adjusted *P* value of less than 0.1.

#### Comparison with a human lung cell data set.

Curated data of control and fibrotic lung cells as published by Habermann et al. (5) were downloaded (GSE135893). A subset of the data was generated to include relevant cell types (mesenchymal and EC from IPF patient lungs or control donor lungs: smoker reject). This reference set of cells was reprocessed via the default SCTransfrom workflow in parallel to the subset of vehicle-only cells from the coculture assay (“query”). Mappings of relevant Habermann et al. (5) cell identity labels to proposed consensus re-annotation of integrated single-cell transcriptomic human lung cell atlas (49) are listed in Supplemental Table 11. For reference and query, we determined the 8,000 most variable genes and determined the union of overlapping variable genes that were mutually expressed in the reference and query data. Average per-cell log expression was calculated for each cluster and used to determine the reported Pearson’s correlations.

For the implementation of SingleR (34), we computed the top 100 positive marker genes for each reference cluster using a Wilcoxon rank-sum test. The query cells were individually profiled against this reference model using SingleR default settings. The per-cell gene module coexpression scores were calculated using Seurat’s “AddModuleScore” function using an approach implemented before (36).

### Data and materials availability

All data needed to evaluate the conclusions in the paper are present in the paper and/or the supplement. RNA-Seq data were deposited in the ArrayExpress database at EMBL-EBI (https://www.ebi.ac.uk/biostudies/arrayexpress) under accession numbers E-MTAB-11832 and E-MTAB-11847. The R command line functions and arguments that were specified for the analysis of the RNA-Seq and sc-RNA-Seq data are listed in Supplemental Table 12. The CellProfiler pipeline to process confocal images is available as Supplemental Data 1.

### Statistics

Calculations were performed using Microsoft Excel. Statistical analysis was performed using GraphPad Prism 6 or R 3.6.0. Data were analyzed using 1- or 2-way ANOVA followed by post hoc tests, according to the experiment (see figure legends for details). A *P* < 0.05 was considered significant.

### Study approval

Formalin-fixed, paraffin-embedded sections of human lungs with IPF or controls (obtained from areas distal to lung cancer resection) were obtained from extra material from patients undergoing surgical lung biopsy for their respective condition at the University of Michigan. Samples were deidentified prior to receipt and thus did not require patient consent to obtain. The University of Michigan IRB provided a waiver of consent.

## Author contributions

AS, RL, SLC, FLG, MS, PB, BR, JR, JHB, PR, BS, DF, RS, MBR, MB, ON, UL, and PS contributed to experimental work, as well as data analysis and/or interpretation. PS, FLG, MS, PB, JR, JHB, RS, MBR, and UL designed and led implementation of experiments, analyzed data, and supervised experimental activity. PS conceived and conceptualized the study. ESW provided human patient lung biopsy specimens for ISH analysis. The manuscript was drafted by PS and ON and was reviewed and edited by all other authors. All authors reviewed and approved the final manuscript as submitted.

## Supplementary Material

Supplemental data

Supplemental data set 1

Supplemental table 1

Supplemental table 12

Supplemental table 2

Supplemental table 6

Supplemental table 7

Supplemental table 8

Supplemental table 9

## Figures and Tables

**Table 1:**
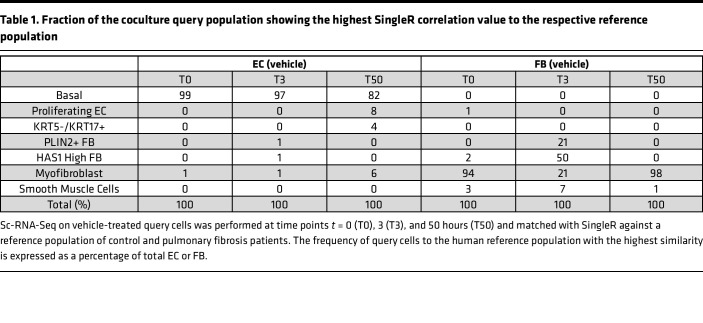
Fraction of the coculture query population showing the highest SingleR correlation value to the respective reference population

## Version 1 12/15/2022

In-Press Preview

## Version 2 02/08/2023

Electronic publication
