# Supplementary material for: NF-κB drives epithelial-mesenchymal mechanisms of lung fibrosis in a translational lung cell model
Source: JCI Insight. 2023 Feb 8;8(3):e154719. doi: 10.1172/jci.insight.154719 (PMC9977429; doi:10.1172/jci.insight.154719)
Supplement: Supplemental data [file jciinsight-8-154719-s046.pdf]

Supplemental Information

NF-κB drives epithelial-mesenchymal mechanisms of lung fibrosis in a translational lung cell model

Patrick Sieber[1]*, Anny Schäfer [1], Raphael Lieberherr[1], Silvia L. Caimi[1], Urs Lüthi[1], Jesper Ryge[1], Jan H. Bergmann[1], François Le Goff[1], Manuel Stritt[1], Peter Blattmann[1], Bérengère Renault[1], Patrick Rammelt[1], Bruno Sempere[1], Diego Freti[1], Rolf Studer[1], Eric S. White[2, ‡], Magdalena Birker–Robaczewska[1], Maxime Boucher[1], and Oliver Nayler[1]

Methods

Cells

Normal human lung fibroblasts (FB) (NHLF; Lonza, CC–2512) and IPF patient–derived FB (IHLF; Asterand, 16769) were used between passage 4 and 8 and cultivated in FB growth medium 2 (FGM–2; Lonza), supplemented with 100 units/ml of penicillin and 100 µg/ml of streptomycin, following the supplier's instructions. Normal human bronchial epithelial cells (EC) (i.e. NHBE; Lonza, CC–2541), NHBEC (Lonza, CC–2540) or HBEpiC (ScienCell, 3210–1ea), as well as human tracheal EC (HTEC; Cell Applications, 06090742) were used between passage 3 and 4 and cultivated in bronchial epithelial cell growth medium (BEGM; Lonza) supplemented according to the supplier’s instructions. The NHBE and HBEpiC were used interchangeably in co–culture with comparable effects and are abbreviated as NHBE in the text for simplicity. The human lung epithelial cell lines NCI–H2452 (ATCC, CRL–5946), A549 (ATCC, CCL–185), NCI–H1975 (ATCC, CRL–5908) and NCI–H1650 (ATCC, CRL–5883) were cultured in RPMI 1640 (Gibco) supplemented with 10% (vol/vol) heat–inactivated FBS (Gibco) and 100 units/ml of penicillin and 100 µg/ml of streptomycin. Human lung epithelial cell lines Calu3 (ATCC, HTB–55) and A427 (ATCC, HTB–53) were cultivated in MEM Eagle medium (Gibco) supplemented with 10% (vol/vol) heat–inactivated FBS (Gibco), 2 mM L–Glutamine, 0.1 mM non–essential amino acids (NEAA), 1 mM Sodium Pyruvate and 100 units/ml of penicillin and 100 µg/ml of streptomycin. Human umbilical vein endothelial cells (HUVEC; Cascade Biologics,) were used at passage 8 and grown in Medium 200 (Cascade Biologics) supplemented with low serum growth supplement (Gibco, S–003–10) and 100 units/ml of penicillin and 100 µg/ml of streptomycin following the supplier's instructions. Normal human bronchial smooth muscle cells (BSMC; Lonza, CC–2576) and human pulmonary artery smooth muscle cells (PASMC; Cell Applications, 352–05a) were used between passage 4 and

8 and cultivated in smooth muscle growth medium 2 (SmGM–2; Lonza), supplemented with 100 units/ml of penicillin and 100 µg/ml of streptomycin, following the supplier's instructions.

Phenotypic HCA

For the high–content assay (HCA), NHLF and NHBE were seeded into standard cell culture vessels 5 days prior to the experiment using their appropriate complete media. Each well of a 384–well clear bottom microtiter plate (Cell Carrier Ultra; Perkin Elmer) was coated with 0.1% laminin solution for 60 min at 37 °C and rinsed once with 1 x phosphate buffered saline (PBS, Gibco). NHBE were stained with CellTracker^{TM} Deep Red (Thermo Fisher Scientific) diluted in 1 x PBS according to the manufacture's protocol. NHLF cells were seeded at a density of 2000 cells/well in a volume of 20 µl FB basal medium (FBM, Lonza) supplemented with 0.1% fatty acid–free BSA, 100 units/ml of penicillin and 100 µg/ml of streptomycin and 250 ng/ml amphotericin B in combination with 800 cells/well NHBE in 20 µl starvation medium (FBM; Lonza) and grown for 48h at 37 °C, 5 % CO_2 and 95 % relative humidity. Control NHLF were grown in complete FB growth medium (FGM–2; Lonza) and differentiated into myofibroblast by incubation with 5 ng/ml TGF–ß1 (from a stock of 20 µg/ml in 4 mM HCl, 1 mg/ml BSA) for 48 h. The final DMSO concentration in the assay was 0.6 % in all wells. 96 hours after seeding the medium was removed, and the cells were fixed in methanol for 10 min at room temperature (RT). After fixation, cells were washed three times with 1 x PBS and either stored at 4 °C for up to 3 days or further processed. The fixed cells were blocked with 10 % goat serum in 1 x PBS, 0.25 % Tween–20 for 60 min at RT. The primary antibody, i.e., mouse monoclonal anti–α–SMA (Sigma–Aldrich; A2547), was diluted 1:400 in 10 % goat serum (Invitrogen) in 1 x PBS, 0.25 % Tween–20 and the fixed cells were incubated with the antibody for 60 min at RT. After washing the cells three times with 1 x PBS, 0.25 % Tween–20 the detection antibody, i.e., goat anti–mouse AlexaFluor 488 (Invitrogen; A11029), was diluted 1:1000 in 1 x PBS, 0.25 % Tween–20 and incubated for 1h at RT together with 1 µg/ml DAPI to stain the nuclei. The stained cells were washed three times with 1 x PBS containing 0.25 % Tween–20, followed by three washes in 1 x PBS, sealed with backing tape and stored at 4 °C. Images

were acquired on the Opera Phenix™ confocal high–content screening system (HCS, Perkin Elmer) using the 20–x water immersion lens.

Image analysis: The image data acquired with the HCS system were uploaded to ORBIT. ORBIT consists of several parts; one part is an open-source image analysis software developed at Idorsia Pharmaceuticals Ltd (www.orbit.bio). The images were analyzed in a highly distributed way using AWS Batch by applying a CellProfiler pipeline. DAPI stained nuclei were used to identify cells. α–SMA regions were then identified and mapped to the corresponding nuclei. After cell segmentation over 400 features, based on the stained cells, were calculated for each cell by CellProfiler as described (1). The computed features comprise granularity, intensity, texture, area, and shape of a cell in α–SMA–expressing regions. NHLF grown as monocultures in dedicated wells on each assay plate were used to define 0% effect controls, i.e. undifferentiated FB. TGF– ß1–mediated fully differentiated myofibroblast were used to define 100% effect controls. These controls were used to train and validate a support vector machine (SVM), a supervised learning model, which was then used to classify every NHLF as either FB or myofibroblast phenotype. The classification of each cell was aggregated to a cell type ratio per well, represented as the computed percentage effect value per well indicative of the degree of myofibroblast differentiation. The pipeline to process the images is available as Supplemental File 1.

Co–culture and protein quantification assay

The following reagents were used for the RapidFire™ MS/MS experiments: 96–well flat bottom culture plate (Corning), 96–deep well plate and 96–well skirted polypropylene polymerase chain reaction (PCR) plate (Greiner), Oasis HLB–well plate 30 µm (5 mg sorbent/well, Oasis), Dulbecco's phosphate–buffered saline (D–PBS, lacking Ca^{2+}/Mg^{2+}), 10 x solution of HEPES and trypsin/EDTA (Invitrogen), tris hydrochloride (Tris–HCl, Applichem), benzonase nuclease (Sigma Aldrich), Tris (2–carboxyethyl) phosphine hydrochloride (TCEP, Sigma Aldrich), iodoacetamide (Sigma Aldrich), LC/MS grade formic acid 50 % (Sigma Aldrich), methanol and acetonitrile CHROMASOLV Plus for HPLC (Sigma Aldrich), urea BioXtra (Sigma Aldrich), as

well as thiourea ACS reagent (Sigma Aldrich). Complete^{TM} Mini EDTA–free protease inhibitor tablets (Roche), ammonium bicarbonate (Fluka), sequencing grade modified trypsin. HPLC plus water (Sigma Aldrich).

NHLF/NHBE co–culture: NHLF were resuspended in FGM–2 growth medium (Lonza), mixed with an equal volume of NHBE in BEGM growth medium (Lonza), and seeded into a 96–well flat–bottom culture plate (Corning) at a density of 20000 and 1600 cells per well, respectively. Compound dilution series were added, and the co–culture was incubated for 18h at 37°C/5 % CO2. The medium was then replaced with FBM (Lonza) containing 0.1 % fatty acid–free BSA and the compounds at the indicated concentrations. The medium was supplemented with 100 units/ml penicillin, 100 µg/ml streptomycin for the entire duration of the culture. If not indicated otherwise the co–culture was incubated at 37°C/5 % CO2 for total 96 h.

Lysis of cells for MS/MS analysis: The cell culture medium was removed, and cells were lysed on ice by adding 5 µl/well of pre–cooled protein extraction buffer containing 10 mM Tris–HCl (pH 8.0), 6 M urea and 2 M thiourea and shaking on ice on a plate shaker at 600 rpm for 30 min. Samples were prepared for MS/MS and surrogate tryptic peptides were chosen for detection of COL1 (COL1A1), α–SMA (ACTA2) and tubulin (TBA1A1) as described (1). Peak areas for COL1A1 and ACTA2 were normalized by dividing by TBA1A1 peak area.

Cell sampling by flow cytometry

For each condition, 5 million NHLF cells were pre–stained with CellTrace^{TM} Far Red (Invitrogen,) and the 1 million NHBE cells with CellTrace^{TM} Violet (Invitrogen) according to the manufacturer's instructions, seeded, either separately or in combination, in full growth medium, at t = 0h and switched to starvation medium at t = 18h, then, at the indicated times after seeding, washed with 1x PBS, trypsinized and resuspended in autoMACS Rinsing Solution (Milteny Biotec) amended with 0.5% fatty acid–free BSA, forced through a 35 µm cell strainer (Corning) to achieve a single cell suspension, FACS sorted, using a cell sorter (SONY, SH800SE) with a 100 µm sorting chip (SONY, LE–C3100), followed by lysis in lysis buffer

(Norgen Biotek). Cells were sorted first based on forward – (FSC) and side scattering (SSC) thresholds, and then gated on CellTrace™ Far Red (638/660nm ex/em) and CellTrace™ Violet (405/421nm ex/em) staining intensities, respectively.

Gene expression analysis by qRT–PCR

mRNA from NHLF, NHBE or FACS sorted co–cultured cells was isolated and reverse transcribed using the Cells–to CT protocol according to the manufacturers' protocol (Thermo Fisher Scientific). QPCR was performed on a Biomark HD (Fluidigm) using 96*96 dynamic arrays using TaqMan assays (Supplemental Table 10, Thermo Fisher Scientific). 18S was used as reference gene after selection from 7 reference candidates (18S, B2M, GUSB, HPRT1, PGK1, PPIA,YWHAZ) based on GENORM evaluation (2). Results were calculated using a modified delta delta cT method where an expression value of 1 reflects no detectable expression. This method allows a comparison between gene expression values for different genes, but also inter–cellular expression based on an identical linear scale. The data are shown as expression values for each gene in NHLF/NHBE from monoculture and NHLF/NHBE from co–culture which were FACS separated prior mRNA isolation.

Gene expression analysis by bulk RNAseq

Total RNA extraction: Cells were isolated by FACS and lysed in RL Lysis Buffer from NORGEN Single cell RNA purification kit (Norgen). Total RNA was isolated using NORGEN Single cell RNA purification kit including a DNase treatment (Norgen), according to the manufacturer's instructions. Quality control was performed using Qubit 3.0 and RNA HS kit (Thermo Fisher Scientific). Quality was assessed with RNA PicoLabChip (Agilent) on a Bioanalyzer. In all samples the ratio of absorption at 260 nm/280 nm was between 1.8 and 2.2 and the RNA Integrity Number (RIN) was > 8.00. All samples passed the quality thresholds.

RNA–seq Library: RNA–Seq libraries of poly(A)–selected RNA were isolated from 50 ng of total RNA using NuGen Universal Plus mRNA–Seq kit (NuGen) according to the manufacturer's recommendations. Final library concentration was determined with Qubit HS

cDNA kit (Thermo Fisher Scientific) and quality was assessed with the Fragment Analyzer HS NGS kit (Agilent). Evaluation of the size, was performed based on a smear range of 100–1000 bp with average size around 330 bp. In parallel qPCR quantification was performed using KAPA library quantification kit (Roche).

RNA–seq sequencing: For the sequencing runs the samples were randomized across two flow cells and sequenced using NextSeq 500/550high Output Kit v2.5 with 75 Cycles (Illumina).

RNA–seq data processing: Sequenced reads were aligned with STAR (v2.5.4b) to the human reference genome (GRCh38) and gene–wise alignments were quantified with featureCounts using *Ensembl* gene annotations. All samples passed quality control (QC) with more than 20 million reads and over 90 % mapping rates. Good on–target (within annotated exons) mapping rates were observed for all samples (more than 80 %). Genes expressing less than 0.58 CPM (corresponding to a minimum of 10 reads) in less than 3 samples (smallest group size) were filtered, leaving 18380 genes in the final count table. Differentially expressed genes were evaluated in R (v4.0) using edgeR (3, 4) for each cell type and condition across time (i.e. NHLF, NHLF-CC, NHBE, NHBE-CC), where genes with an FDR < 0.05 and a linear fold change (linFC) > 1.5 were considered significant. Expression levels per gene were calculated as transcripts per million (TPM), defined as the number of aligned reads divided by gene length followed by normalization with library size (i.e. total number of on-target reads per sample divided by 1e6). Low expressed genes were filtered from the final DEG list, such that genes with an expression (TPM) below 1 across all samples were excluded from subsequent analysis (e.g. clustering).

Gene expression signatures and their transcriptional regulation: Lists of differentially expressed genes (DEG) among cell types were compared using Venn diagrams to distinguish specifically or commonly regulated genes. The resulting gene signatures were uploaded into Ingenuity Pathway Analysis (IPA, Ingenuity System, Qiagen) application for further biological interpretation.

Upstream Regulator Analysis, as implemented in the IPA software (Ingenuity System, Qiagen) and based on expected causal effects between upstream regulators and targets, was used to identify upstream regulators (separated by a "single hop"), that may be responsible for gene expression changes observed in the experimental dataset. IPA predicts which upstream regulators are activated or inhibited to explain the up–regulated and down–regulated genes observed in the dataset. An absolute z–score of ≥ 2 is considered significant. Predictions where both the z-score (absolute value > 2) and the p–value (Fischer's exact test) was significant (< 0.001) were considered reliable predictions.

Time series clustering: Significantly expressed genes within each cell type (NHFL: 4877; NHLF–CC: 6289; NHBE: 6090; NHBE–CC: 6205) were clustered across time using a noise–robust soft clustering method based on the fuzzy c–means algorithm implemented in the R–package Mfuzz (5, 6). Gene expression values were converted to z–scores prior to clustering (for each gene, subtracted mean and divided by SD) using the function standardize() in the Mfuzz package. The number of time series clusters per cell type were evaluated by hierarchical clustering on the Euclidean distance between sample expression z–score vectors using Ward agglomeration method. Overlap between time series clusters of the different cell populations were illustrated using the R package UpSetR (7), an extension of Veen diagrams for visualization of overlapping sets.

Gene Set Overexpression Analysis (GSOA): For each cell type, GSOA was performed for each time series cluster using the R package hypeR (8) with MSigDB (9, 10) and Enrichr (11, 12) gene sets.

Single cell library preparation and sequencing

NHLF and NHBE were combined at a ratio of 5 : 0.4 in full growth medium (FGM–2 and BEGM (1:1 vol./vol.; Lonza). After 18h medium was exchanged for FBM starvation medium (Lonza). The medium was supplemented with 100 units/ml penicillin, 100 µg/ml streptomycin for the entire duration of the culture. The co–culture was then incubated at 37°C/5% CO_2. After 3h,

and 50h, respectively, growth medium was aspirated and co–cultured cells in monolayer were gently detached using 0.05% trypsin/EDTA. Cells were resuspended in ice–cold FACS buffer (autoMACS Rinsing Solution, Miltenyi Biotec) supplemented with 0.5% fatty acidCfree BSA (Calbiochem) to approximately $1x10^6$ cells/ml. Time point 0h cells were combined directly in ice–cold FACS buffer. Viability was determined using Trypan blue staining on an automatic Vi–Cell Analyzer. Average viability of all samples taken into single cell sequencing was 92.5% (88.4% – 94.8%). Cell suspensions were immediately loaded on a Chromium Next GEM Chip K (10X Genomics) targeting 10000 cells per channel for recovery. Single cell encapsulation and RNA capture was controlled on the Chromium Single Cell Controller. Single cell libraries were prepared using the Chromium Next GEM Single Cell 5' Kit v.2 reagents according to the manufacturer's recommendations. Final libraries were pooled and sequenced to an average depth of >42000 reads per cell on an Illumina NovaSeq system by Genewiz (Germany).

Single cell RNA–seq preprocessing

Gene by cell counts matrices were processed from raw fastq files using Cellranger count (v4.0.0) by mapping against the human GRCh38 reference. The filtered gene matrices were loaded and further processed using Seurat v3.9.9.9002. As part of our stringent quality filtering, cell doublets were identified using scDblFinder(13) (scDblFinder: R package version 1.4.0, https://github.com/plger/scDblFinder) and removed. Additionally, we removed cells with > 15% mitochondrial reads or with > 60% ribosomal protein–coding gene reads. Low complexity cells with a log (genes per UMI) ratio of less than 0.8 were discarded. We further defined inclusion thresholds based on RNA molecule counts and detected genes count.

In brief, we used SCTransform (14) implemented in Seurat to perform per–sample normalization. As we noticed a pronounced effect of cell cycle phase in the first dimensions of a principal component analysis (PCA), we further regressed out cell cycle effects based on G2/M– and S–phase scores calculated in Seurat from a set of known cell cycle phase marker genes as described in Tirosh et al. (15). Normalized datasets were integrated using Seurat's anchor–finding workflow based on canonical correlation analysis (16). A first–level graph–

based clustering was performed on the full integrated dataset using the first 30 PCA dimensions for neighbor–finding and subsequent Louvain clustering using a resolution of 0.2. Choice of the resolution parameter was guided by assessing cluster partitioning using the clustree package (17). For visualization, UMAP embeddings were calculated based on the first 30 PCA dimensions. EC populations were determined based on known marker gene expression (*CDH1*, *EPCAM*, *TP63*) and used for sub–clustering analogous to the above workflow, using the first nine PCA dimensions and a resolution parameter of 0.25. Sub–cluster groups were reverse–annotated to refine the epithelial first–level clusters and projected into the same UMAP coordinates for visual consistency.

For labeling of clusters, we used a Wilcoxon rank sum test implemented in Seurat's FindConservedMarkers function to determine cluster–specific marker genes independent of timepoint or treatment. Test requirement was set to a minimum log–fold change of 0.25 and detection in at least 20 % of cells in a given cluster from up to 500 cells per cluster. Expression testing was performed on the log–normalized counts. Gene pathway assessment of markers with a Bonferroni–adjusted p value of less than 0.01 was performed using GSEA (http://www.gsea–msigdb.org).

Single cell RNA–seq analysis

Differential state analysis: A random forest classifier to predict culture time effect for each cell cluster group was built and tested using Augur (18) and default settings providing log–normalized RNA assay as input. Only clusters with a minimum of 20 cells were tested.

Pair–wise differential expression across timepoints or treatments was performed using MAST (19) implemented in Seurat after merging all EC into one group. Differential testing was performed for clusters with at least 40 cells total and a minimum of 10 cells per condition. Only genes detected in at least 15 % of cells in a cluster group and a minimum log–fold change of 0.25 were tested. Expression testing was performed on the log–normalized counts. Results were filtered based on a Bonferroni–adjusted p–value of less than 0.1.

Pseudotime: Trajectory analysis was performed on the clustered vehicle group samples using the R package slingshot (v1.8.0)(20) on dimensionality reduced expression values (UMAP). The analysis was performed separately on EC and FB, using EC_FBXO2 and FB_APOE respectively as seed clusters. In addition, the Monocle3 (1.0.0)(21) R package was used to generate a more refined trajectory between cell states.

Pathway enrichment analysis: GSOA on DEGs was performed using the R package hypeR (8) querying the MSigDB (9, 10) conserved pathways (CP) collection including the REACTOME – and BIOCARTA collection, the gene ontology (GO) collection with biological process (BP) –, cellular component (CC) –, and molecular function (MF) collection, as well as the Hallmark (H) collection gene sets, respectively. Gene sets with a FDR < 0.05 were deemed significant.

Cell–Cell Interactions: CellphoneDB (3.0.0)(22) was used to analyze cell–cell interactions. The analysis was performed separately on each sample (time point and condition). From the resulting interactions, the ligand receptor pairs were extracted omitting integrins and filtered such that only unique epithelial–FB interactions were retained. These interactions were visualized using Cytoscape(23).

Comparison with a human lung cell data set

Curated data of control and fibrotic lung cells as published by Habermann et al (24) was downloaded (GSE135893). A subset of the data was generated to include relevant cell types (mesenchymal and EC from IPF patient or control donor (smoker reject lungs). This "reference" set of cells was re–processed via the default SCTransfrom workflow in parallel to the subset of vehicle–only cells from the co–culture assay ("query"). For each, reference and query, we determined the 8000 most variable genes and determined the union of overlapping variable genes that were mutually expressed in the reference and query data. Average per–cell log expression was calculated for each cluster and used to determine the reported Pearson correlations.

For the implementation of SingleR (25), we first trained a prediction model from the reference data counts, using all genes mutually detected in the reference and query subsets. For each reference cluster, we computed the top–100 positive marker genes using a Wilcoxon rank sum test. The query cells were individually profiled against this reference model using SingleR default settings. The reported prediction score corresponds to the 0.8 quantile of Spearman correlations for each cell across all reference cell types. The per–cell gene module co–expression scores were calculated using Seurat's "AddModuleScore" function using an approach implemented in (26). The score represents the average expression of the n module genes, subtracted by a control score for n * 100 randomly selected genes with comparable expression.

RNAscope in situ hybridization

Formalin–fixed, paraffin–embedded sections of human lungs with IPF or controls (obtained from areas distal to lung cancer resection) were obtained from extra material from subjects undergoing surgical lung biopsy for their respective condition at the University of Michigan. Samples were de–identified prior to receipt and thus did not require patient consent to obtain. RNAscope and immunofluorescence staining were performed as described (27, 28) using the following probes: Homo sapiens collagen type 1 alpha 1 (*COL1A1*) mRNA (Cat. No. 401898), Homo sapiens integrin subunit alpha V (*ITGAV*) transcript variant 1 mRNA (Cat. No. 488398), Homo sapiens integrin subunit beta 6 (*ITGB6*) mRNA (Cat. No. 312498). A digital quantification of the mRNA staining was performed using Orbit Image Analysis software, version 3.71(29). The nuclei and the mRNA signal areas were annotated manually. Supervised segmentation models of the annotated image structures were created and used to train a support vector machine classification model. Using a custom script in Orbit, the images were divided into 13.9µm x 13.9µm (64 x 64 pixels) regions. For each region, the ratio between the area of mRNA expression (red dots) and the area of the nuclei was formed and binned into 256 categories representing the signal intensity levels from 0 (no expression) to 255 (very strong expression). To study the co–localization of the mRNA staining across

different tissue sections, the images were registered using the elastix framework [https://www.bibsonomy.org/bibtexkey/journals%2ftmi%2fKleinSMVP10/dblp]. The slide images showing the *COL1A1* expression were used as still reference images and the ones showing the expression of *ITGAV*, and *ITGB6*, were warped to align with the reference. B–spline transformations were computed to align with local changes. To provide an insightful rendering of the quantifications, the calculated signal intensities were further divided into coarser regions. In the case of the *COL1A1* gene expression the averaged signal intensity within 8 x 8 of the regions was rendered, while the maximum signal intensity of the *ITGAV* and *ITGB6* expression was rendered.

Cell viability and cytotoxicity assay

CHO–K1 cells (ATCC, CCL–61) were seeded, at a density of 20000 cells per well in a tissue culture–treated 96–well flat bottom culture plate (Costar) in Ham's F–12 (Gibco) supplemented with 10% non–heat–inactivated FBS (Amimed) and 1% Penicillin/Streptomycin (Gibco), with compounds at the indicated concentrations and incubated with the CellTox™ Green Cytotoxicity Assay (Promega) and RealTime–Glo™ MT Cell Viability Assay (Promega) reagents following the recommendations of the manufacturer. The fluorescence–associated membrane integrity (a surrogate for cytotoxicity) was calculated from the measured fluorescence (excitation/emission wavelength: 485 nm/530 nm), expressed as relative fluorescent units (RFU). The background fluorescence (cell–free wells; 0 % CellTox) was subtracted and the difference was expressed as a percentage in comparison to cells displaying maximum cytotoxicity (after addition of cell lysis buffer, 100 % CellTox). The luminescence–associated metabolic activity (a surrogate for cell viability) was calculated from the measured luminescence, expressed as relative luminescence units (RLU). The background signal (cell–free wells; 0 % viability) was subtracted and the difference was expressed as a percentage of the maximum metabolic activity (DMSO–treated cells, 100 % metabolic activity).

Figures

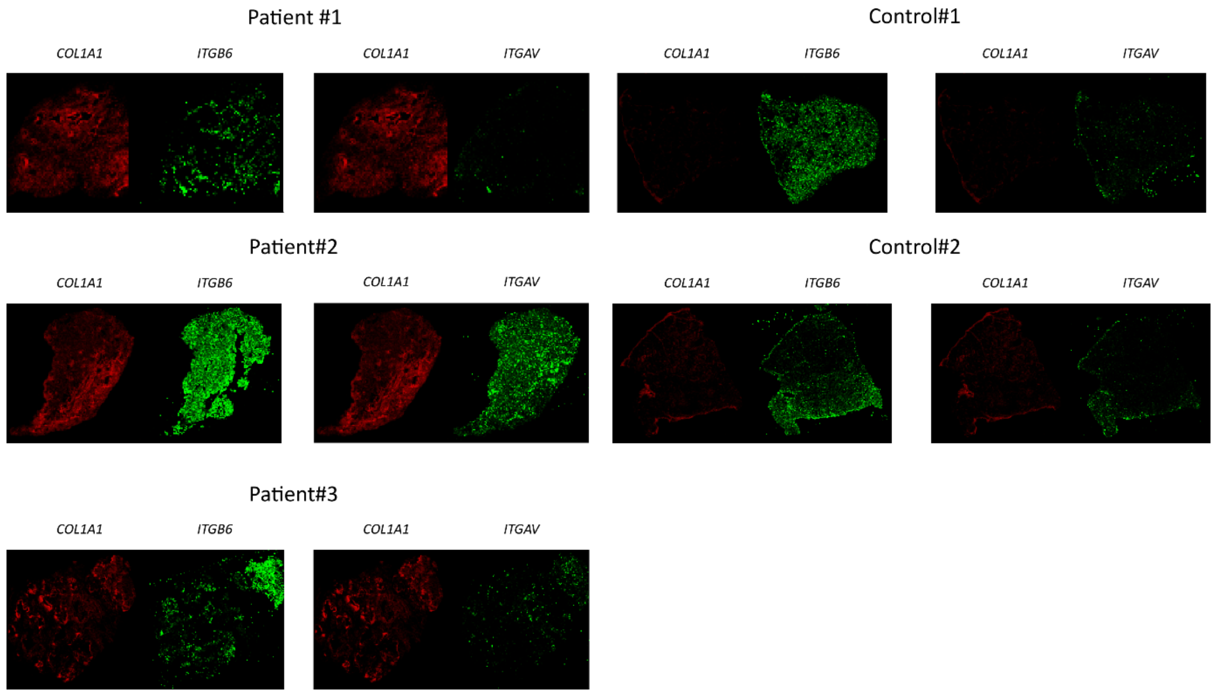

Supplemental Figure 1. Expression of *COL1A1*, *ITGB6*, and *ITGAV* on computer–aligned RNAscope–ISH overview images of lung sections. Near consecutive tissue sections showing the expression of epithelial marker genes integrin subunit beta 6 *(ITGB6)* and integrin subunit alpha V *(ITGAV)*, as well as the FB/myofibroblast marker collagen type 1 alpha 1 *(COL1A1)*, as detected by RNAscope ISH on formalin–fixed, paraffin–embedded patient and control lung sections. Images were computationally aligned using Q–path software. For the analyzed genes, the RNAscope ISH signal was quantified for each grid square and projected onto the tissue in false color as intensity. Across the entire lung section, *COL1A1*, *ITGB6*, and *ITGAV* showed overlapping expression. Regions of high expression intensity exhibit a tissue–specific intensity pattern and are more abundant in patients. For each gene, signal per square area (1μm^2) is quantified and expressed as false color intensity. Areas showing high expression are shown as brighter signal intensity. *COL1A1* expression (red), representing the FB/myofibroblast compartment, is shown aligned to the epithelial markers *ITGB6* and *ITGAV* (green), respectively. All images were processed with the same settings.

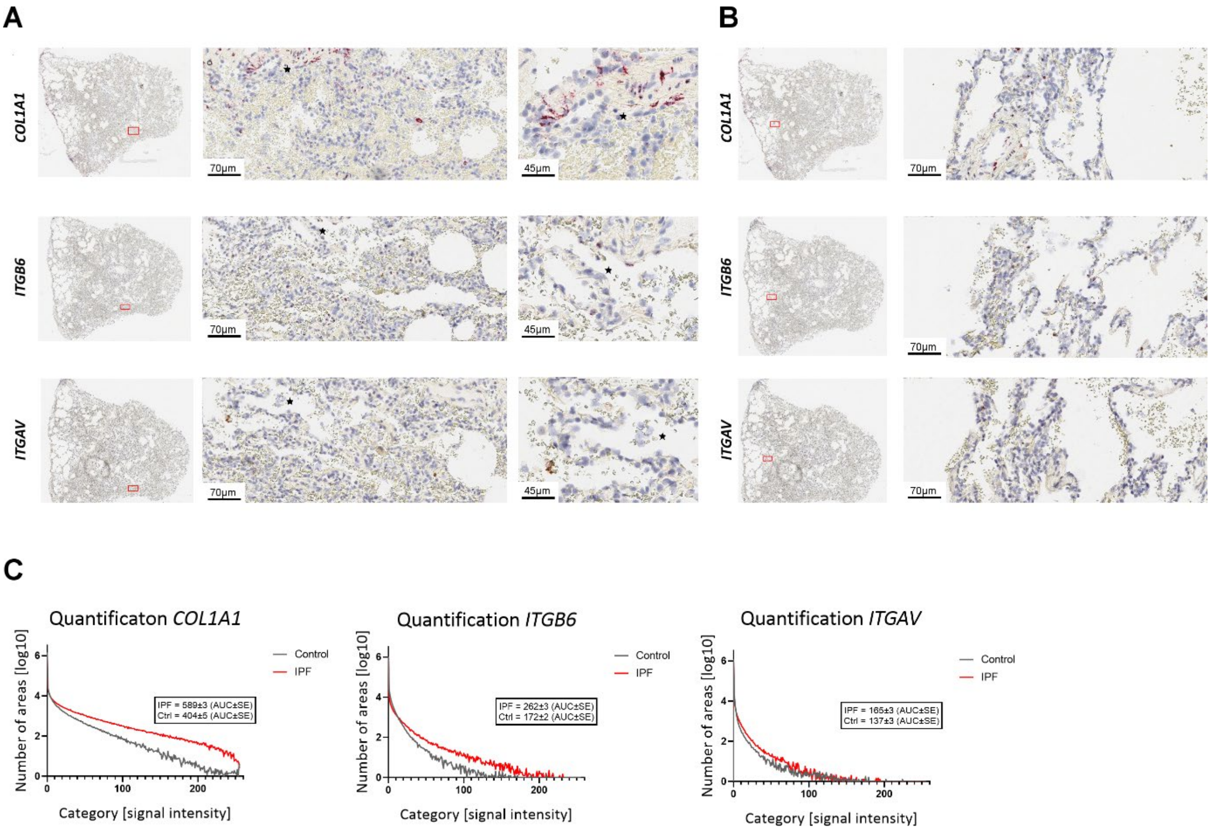

Supplemental Figure 2. Expression of *COL1A1*, *ITGB6* and *ITGAV* in control lungs. (A, B) Expression of *COL1A1*, *ITGB6* and *ITGAV*, as detected by RNAscope ISH on formalin–fixed, paraffin–embedded control lung sections. Boxed area in the low magnification lung section overviews in the left image indicates the enlarged region. The asterisks point to the approximate same location in close consecutive tissue sections. **(A)** A representative section of a denser tissue region and **(B)** a region with preserved alveolar tissue structure is shown. **(C)** Histograms showing the distribution of RNA signal intensities of *COL1A1, ITGB6*, and *ITGAV* in control and IPF patient lungs, as quantified by Orbit. Tissue sections were segmented into areas of equal size, and the number of RNA signals per area was quantified and divided by the nuclear area contained therein to normalize for cell number. Each area was then categorized from 0 (no signal) to 255 (very strong signal intensity) and plotted, as a histogram for each condition. X–axis: number of areas expressed on a log10 scale; Y–axis: graded signal intensity categories. The calculated area under the curve (AUC) represents a measure for the total signal intensity per condition. *N* = 3 samples per condition.

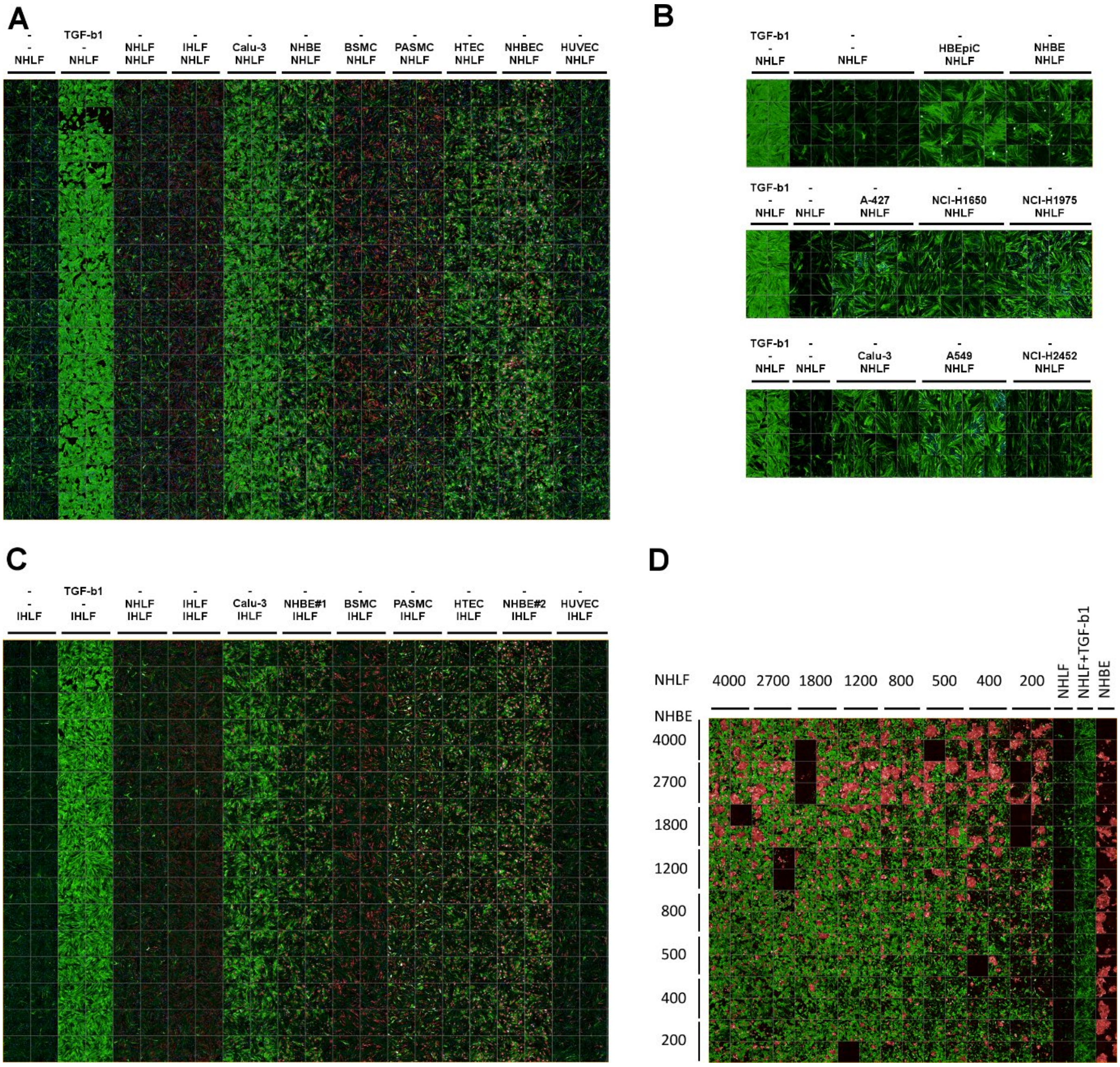

Supplemental Figure 3. EC in co–culture induce NHLF – and IHLF to myofibroblast differentiation. High–content confocal microscopy images of **(A, B)** normal human lung fibroblasts (NHLF) and **(C)** IPF–patient–derived human lung fibroblasts (IHLF), which accumulate α–SMA (green) to variable extent when co–cultured over a period of 5 days with primary normal human bronchial epithelial cells (NHBE; NHBEC), normal human tracheal epithelial cells (HTEC), immortalized epithelial cell lines (Calu–3; A427; A549; NCI–H2452; NCI–1975; NCI–1650), NHLF, IHLF, bronchial smooth muscle cells (BSMC), pulmonary artery smooth muscle cells (PASMC), or human umbilical vein endothelial cells (HUVEC). NHLF cells stimulated with TGF–ß1 (5 ng/ml) served as positive control. 2’000 FB effector cells and 800 stimulating cells per well were seeded, respectively. Stimulating cells were stained with CellTracker Deep Red, nuclei with DAPI, and α–SMA by using an anti–α–SMA antibody. The merged green and red channels are shown. **(D)** NHLF were titrated against NHBE to determine the effect of cell number and cell type ratio at seeding.

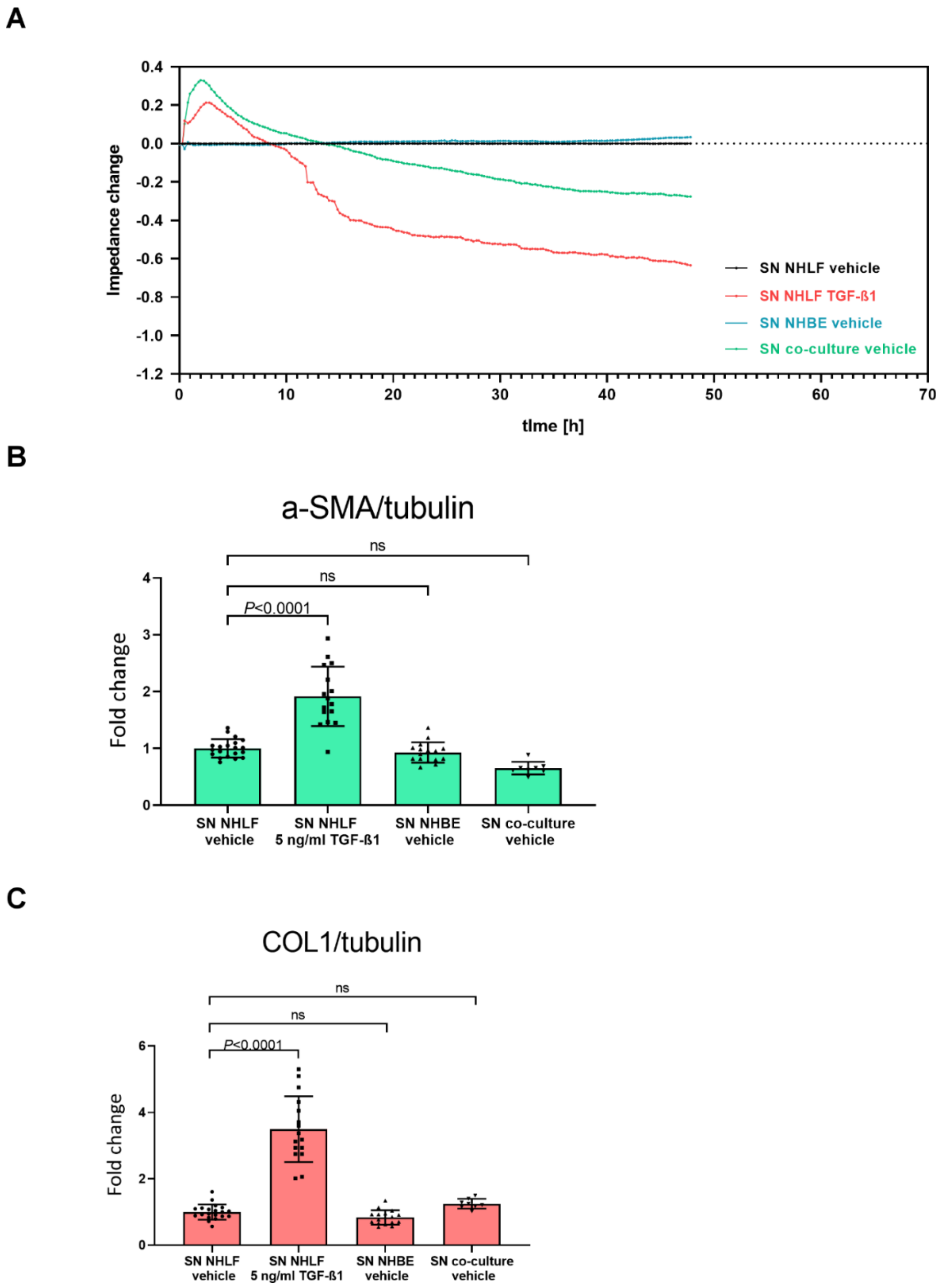

Supplemental Figure 4. Conditioned co–culture supernatant is insufficient to trigger pro–fibrotic changes in NHLF. (A) Impedance recordings of NHLF cells. Cells were seeded in full growth medium. After 18h medium was replaced by starvation medium supplemented with conditioned cell culture supernatant in a 1:1 (vol/vol) ratio. *N* = 8 for each condition**. (B)** α–SMA and **(C)** COL1, normalized to tubulin, were quantified by MS/MS from NHLF cultured with added supernatant of NHLF treated with 0.1% DMSO (SN NHLF vehicle), NHLF treated with 5 ng/ml TGF–ß1 in 0.1% DMSO (SN NHLF TGF–ß1), NHBE treated with 0.1% DMSO (SN NHBE vehicle), and NHLF/NHBE co–culture treated with 0.1% DMSO (SN co–culture vehicle) for 80h, respectively. Bars depict mean ± SD. A one–way ANOVA with Dunnett's multiple comparison test was used. *N* = 16 for each condition.

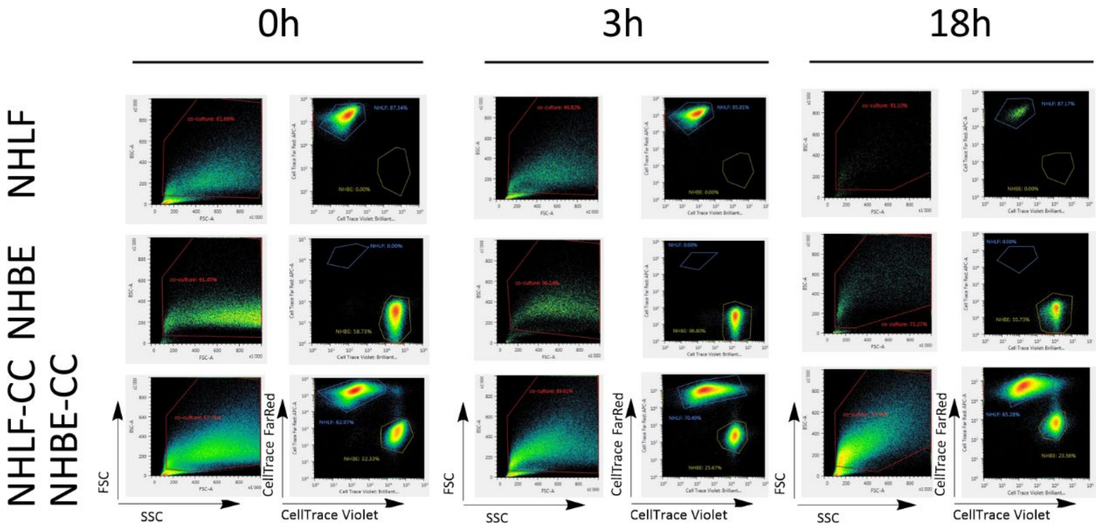

Supplemental Figure 5. Capturing of cells for the analysis of early gene expression changes. For each condition, NHLF cells were pre–stained with CellTrace Far Red and the NHBE cells with CellTrace Violet, seeded, either separately or in combination as depicted, at t = 0h and then FACS sorted, followed by lysis at t = 0h, 3h, and 18h. Left hand charts show scatter plots based on forward (FSC) and side scattering (SSC) profiles of monocultured (NHLF, NHBE) and of co–cultured (NHBE–CC, NHBE–CC) cells, as determined by flow cytometric analysis. Red line indicates gating threshold. Right hand panels: Gated cells were sorted on CellTrace Far Red and CellTrace Violet staining intensities, respectively. Blue and yellow gates indicate relative number of sorted NHLF and NHBE, expressed in (%) of total sorted cells, respectively.

400

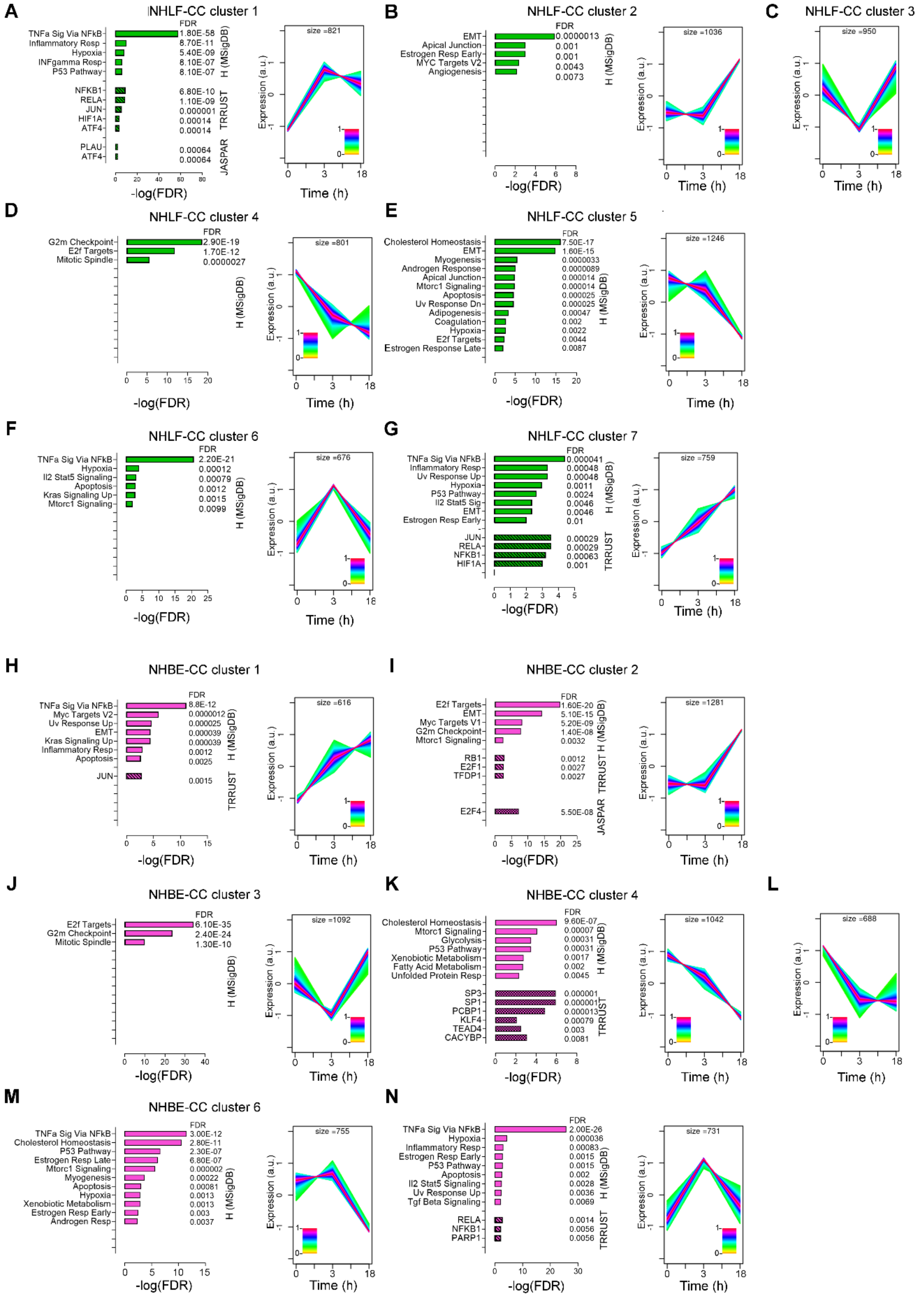

401

402 **Supplemental Figure 6. Gene Set Overexpression Analysis in clusters of co–regulated**
403 **genes in co–cultures of NHLF and NHBE.** 6289 NHLF–CC genes and 6205 NHBE–CC

genes showed a significant (FDR < 0.05 and |LinFC| > 1.5) change in gene expression from t = 0h with detected expression max(count) [TPM] > 1. Based on their gene expression changes over time NHLF–CC and NHBE–CC DEG were each assigned to 7 clusters of comparable size and as determined by the Mfuzz algorithm. **(A-G)** Expression kinetics and results of the gene set overexpression analysis (GSOA) of the seven NHLF–CC clusters and **(H-N)** of the seven NHBE-CC clusters. The comprised expression kinetics of the clustered genes is depicted as a z–score (from –1 to 1; y–axis) across the time points t = 0h, 3h, and 18h, respectively (x–axis). The expression pattern of each gene is associated with a cluster weight between 0 and 1 (according to its match with cluster dynamics), color coded in the figure according to the inserted palette. For each cell type, GSOA was performed for each time series cluster using the R package hypeR querying the MSigDB Hallmark (H) and the JASPAR and TRRUST collections with gene sets provided by Enrichr. Enriched gene sets are displayed as bars representing the –log10(FDR) with the corresponding FDR values.

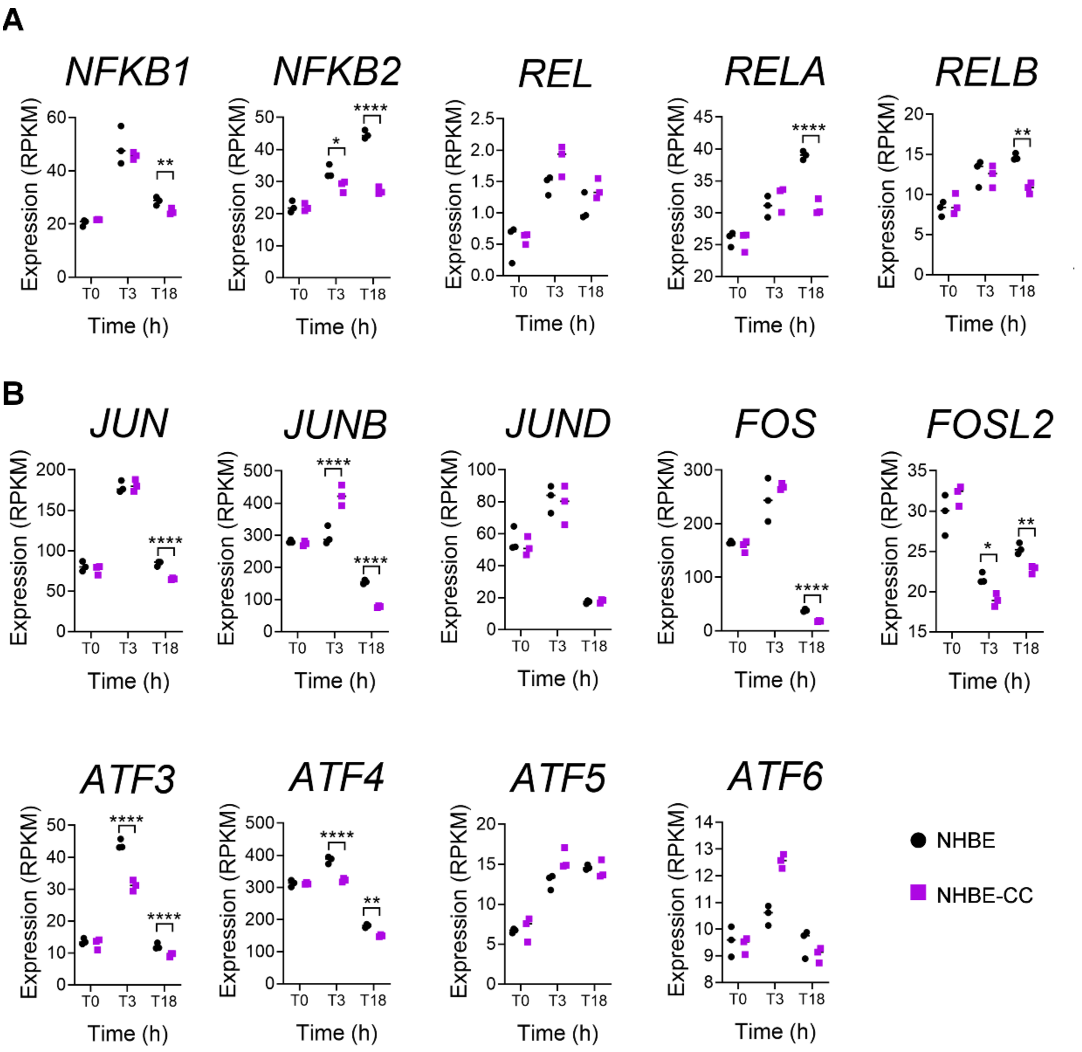

Supplemental Figure 7. Gene expression of NF–κB and AP–1 transcription factor subunits in monocultured and co-cultured NHBE. Gene expression, depicted in reads per kilobase of transcript, per million mapped reads (RPKM), of **(A)** NF–κB and **(B)** AP–1 transcription factor family subunits for the conditions NHBE and NHBE–CC showing gene expression differences between time points t =0h and 3h, or 18h, respectively. Differentially expressed genes in comparison to monocultured control were evaluated using edgeR and are depicted as * FDR ≤ 0.05, ** FDR ≤ 0.01, *** FDR ≤ 0.001, and **** FDR ≤ 0.0001.

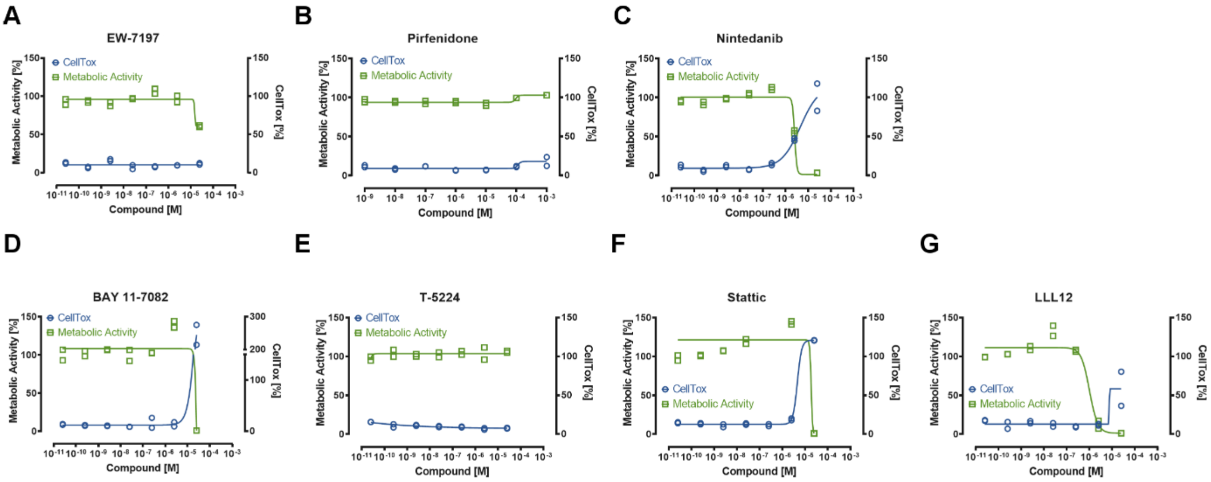

Supplemental Figure 8. Cell viability and cytotoxicity of compounds. Multiplexed viability measurement depicting metabolic activity (RealTime–Glo™ MT) and cytotoxicity / membrane integrity (CellTox™ Green). The compounds **(A)** EW–7197, **(B)** pirfenidone, **(C)** nintedanib, **(D)** BAY 11–7082, **(E)** T–5224, **(F)** LLL12, and **(G)** stattic were added to the CHO–K1 cells at t = 0h covering the concentration range 0.025 nM – 25000 nM, except for pirfenidone which was tested at 0.01 µM – 10000 µM and incubated with the CellTox™ Green – and RealTime–Glo™ MT reagents for 18h. The fluorescence–associated cytotoxicity and the luminescence–associated metabolic activity (a surrogate for cell viability) was calculated as described in the Material and Method section and expressed as a percentage of the maximum cytotoxicity (addition of cell lysis buffer; 100% cell tox) or metabolic activity (DMSO–treated cells, 100% metabolic activity), respectively. 0.25 % DMSO solvent was present in all wells. $N = 2$.

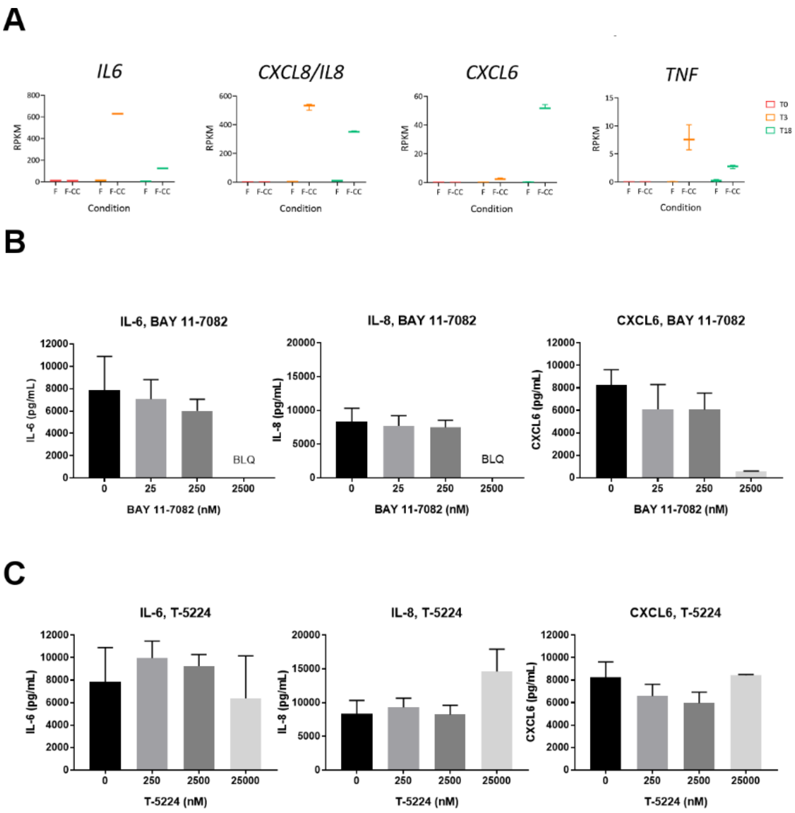

Supplemental Figure 9. The NF–κB inhibitor BAY 11–7082 blocks cytokine production in the co–culture. (A) Box and whisker plot expression, as depicted in reads per kilobase of transcript, per million mapped reads (RPKM), in NHLF (F) and NHLF–CC (F–CC), of *IL6, CXCL8/IL8, CXCL6,* and *TNF* at t = 0h (T0), 3h (T3), and 18h (T18), respectively. **(B)** Effect of the NF–κB inhibitor BAY 11–7082, and **(C)** c–FOS/AP–1 inhibitor T–5224 incubated with the co–cultured NHBE–CC and NHLF–CC from t = 0h until t = 18h, at the indicated concentrations, on levels of secreted IL–6, IL–8 and CXCL6 at t = 98h, as determined by Luminex and depicted in (pg/ml).

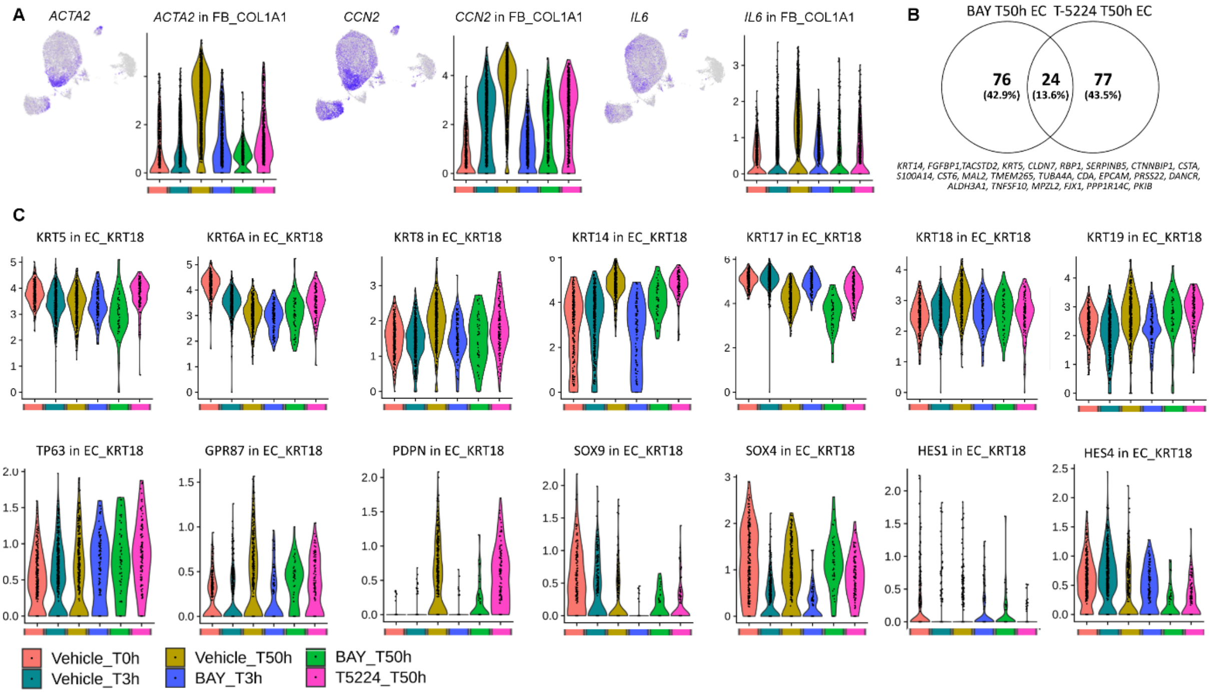

Supplemental Figure 10. Effect of c–FOS/AP–1 –, and IKK/NF–κB inhibition on marker gene expression at sub–population resolution. **(A)** UMAP embeddings of jointly analyzed single cell transcriptomes from 18’676 vehicle control cells collected at the time points t = 0h, 3h, and 50h, respectively, overlayed with the expression (log normalized counts) of *ACTA2, CCN2,* or *IL6* are shown next to violin plots depicting the expression *ACTA2, CCN2,* and IL6, separated by sample, for the cell sub–state “FB COL1A1”. **(B)** Venn diagram representation of epithelial cell genes affected by each treatment a t = 50h. **(C)** Violin plots depicting the expression, separated by sample, of marker genes *KRT5, KRT6A, KRT8, KRT14, KRT17, KRT18, KRT19*, *TP63, GPR87, PDPN, SOX9, SOX4, HES1,* and *HES4* at the level of sub–clustered cell state identifier “EC KRT18”. Cells were either untreated (vehicle) or treated (i.e. 2.5 µM BAY 11–7082, 25 µM T–5224) and collected at the time points t = 0h, 3h, or 50h, respectively. Normalized gene expression is depicted as log(counts+1) for a non–statistical overview of gene–of–interest expression in the dataset.

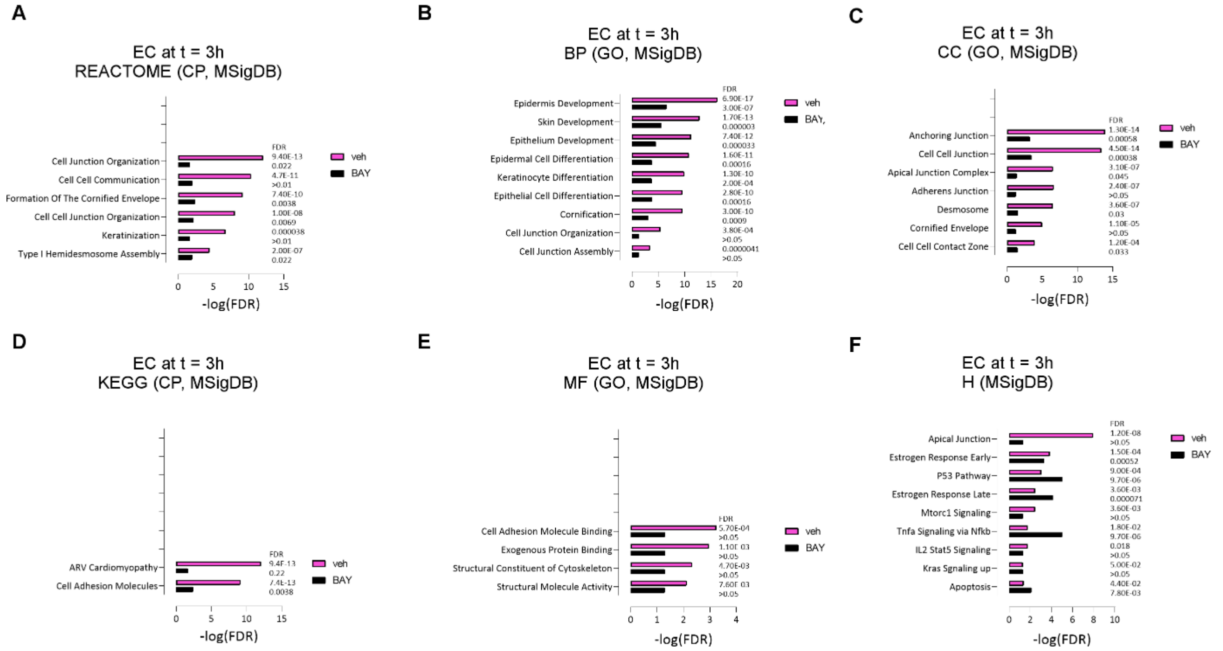

Supplemental Figure 11. Enriched canonical pathways and function in the EC at t = 3h. Effect of the NF–κB inhibitor BAY 11–7082 (2.5 μM) or vehicle control, applied from t = 0h, on overrepresented gene sets in the EC DEG, identified by MAST, at time t = 3h vs t = 0h, displayed as bars representing the –log10(FDR) of the MSigDB data base query results using hypeR.**(A)** conserved pathways (CP); REACTOME –, **(B)** gene ontology (GO); biological processes (BP) –, **(C)** GO; cellular components (CC) –, **(D)** CP; Kyoto Encyclopedia of Genes and Genomes (KEGG) –, **(E)** GO; molecular function (MF) –, and **(F)** Hallmark gene sets.

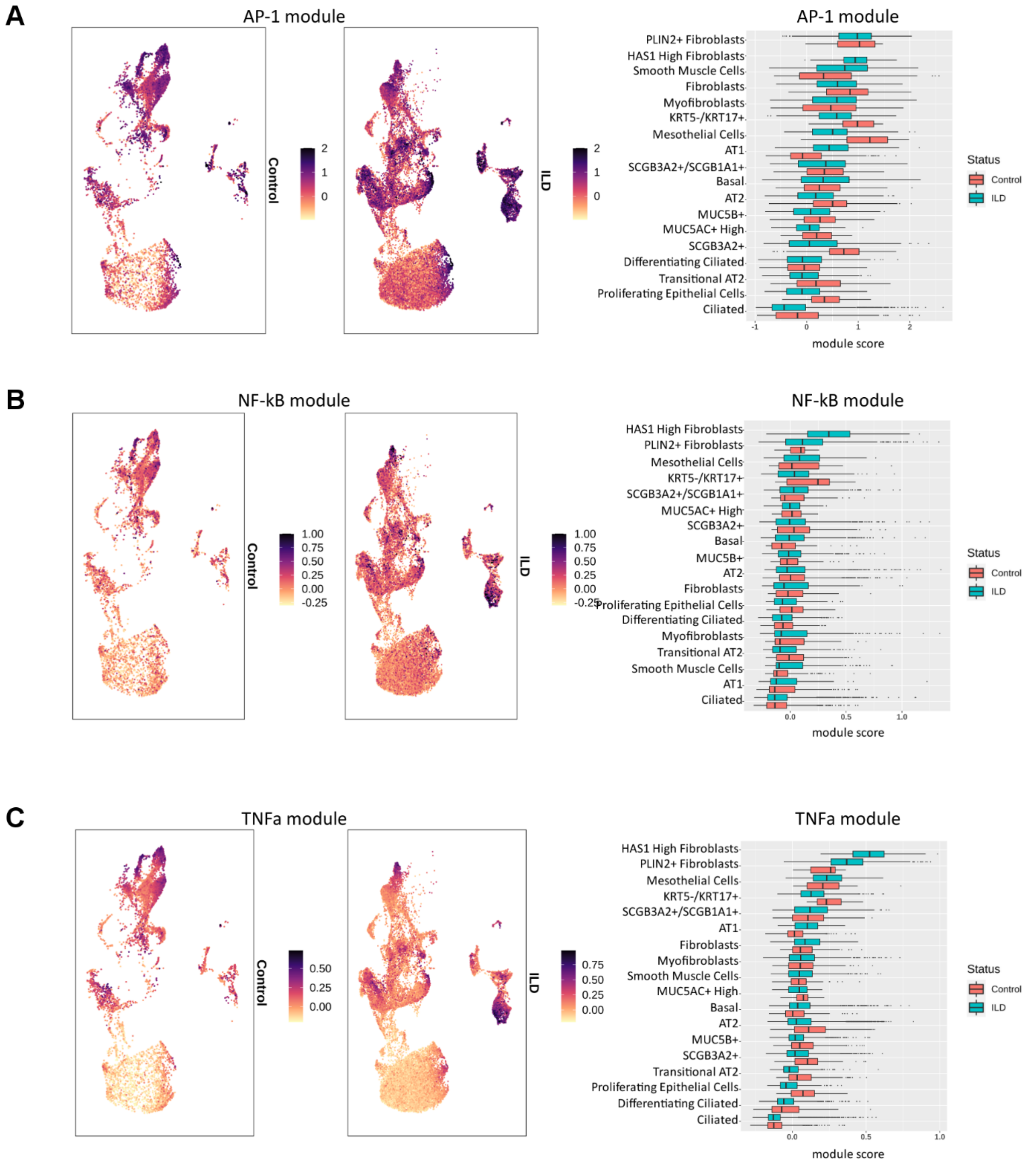

Supplemental Figure 12. Expression of AP–1 and NF–κB gene modules in cell populations isolated from IPF patient lungs. Results of the module score analysis querying the Habermann reference human IPF patient lung cell atlas (24) with the gene expression modules **(A)** AP–1, **(B)** NF–κB, and **(C)** "TNF–α Signaling via NF–κB". The UMAP space of control and interstitial lung disease (ILD) cells is overlaid with the obtained module scores for each individual cell. Box plots display module scores (x–axis) obtained for the respective reference cell populations (y–axis) in control (red boxes) and ILD patient–derived (turquoise boxes) cells.

Tables

Supplemental Table 1. DEG list mono vs co–culture at t = 3h and t = 18h.

Supplemental Table 2. Co-culture specific genes associated with top-ranked canonical pathways

Supplemental Table 3. IPA predicted Upstream Regulators of 1599 NHLF–CC DEG (t = 3h)

Upstream Regulator	Molecule Type	Predicted Activation State	Activation z–score	* p–value
TNF	cytokine	Activated	7.891	1.16E–21
IL1A	cytokine	Activated	5.23	2.51E–16
NFkB (complex)	complex	Activated	7.829	2.86E–15
IL1B	cytokine	Activated	6.884	1.31E–13
CSF2	cytokine	Activated	2.485	1.52E–12
IFNG	cytokine	Activated	4.412	1.16E–11
CHUK	kinase	Activated	5.482	1.43E–11
TP53	transcription regulator	Activated	2.664	2.32E–11
ERK	group	Activated	4.198	3.86E–11
IKBKB	kinase	Activated	5.446	7.72E–11
poly rI:rC–RNA	biologic drug	Activated	5.045	1.75E–10
NOD2	other	Activated	3.449	3.12E–10
ECSIT	transcription regulator	Activated	3.501	1.5E–09
NFKBIA	transcription regulator	Activated	2.792	2.31E–09
CD40LG	cytokine	Activated	3.666	2.34E–09
CDKN1A	kinase	Activated	2.359	2.56E–09
STAT3	transcription regulator	Activated	3.845	3.25E–09
IKBKG	kinase	Activated	4.028	4.39E–09
RELA	transcription regulator	Activated	5.309	4.52E–09
RABL6	other	Inhibited	–2.982	6.87E–09
IL4	cytokine	Activated	2.84	6.93E–09
TNFSF11	cytokine	Activated	4.017	2.33E–08
CD40	transmembrane receptor	Activated	4.916	2.78E–08

CDKN2A	transcription regulator	Activated	3.487	4.81E–08

* Top–ranked. Data were sorted according to the ascending p–value.

Supplemental Table 4. IPA predicted Upstream Regulators of 1364 NHBE–CC DEG (t = 3h)

Upstream Regulator	Molecule Type	Predicted Activation State	Activation z–score	*p–value
miR–369–5p	mature microRNA	Activated	2.111	1.58E–07
CDKN2A	transcription regulator	Activated	2.077	1.27E–05
TNF	cytokine	Activated	2.965	1.97E–05
CCND1	transcription regulator	Inhibited	–2.074	6.98E–05
NUPR1	transcription regulator	Activated	3.175	1.17E–04
RELA	transcription regulator	Activated	2.411	4.88E–04
PDLIM2	other	Inhibited	–2.138	5.24E–04
NFkB (complex)	complex	Activated	2.446	9.37E–04

* Top–ranked; adjusted p–value < 0.001. Data were sorted according to the ascending p–value.

Supplemental Table 5. IC$_{50}$ values for small molecule inhibitors

Compound name	Activity	MS/MS α–SMA IC$_{50}$ [nM]	MS/MS COL1 IC$_{50}$ [nM]
EW–7197	ALK5 inhibitor	17 ± 13 (n = 8)	20 ± 7 (n = 8)
pirfenidone	n.a.	>1’0000’000 (n = 6)	6’577’000 ± 3’860’000 (n = 6)
nintedanib	EGFR/VEGFR/FGFR inhibitor	277 ± 287 (n = 4)	214 ± 70 (n = 6)
BAY 11–7082	IKK/NF–κB inhibitor	415 ± 403 (n = 4)	428 ± 241 (n = 4)
T–5224	c–FOS/AP–1 inhibitor	17930 ± 9366 (n = 4)	10267± 9557(n = 4)
stattic	STAT3 inhibitor	52 ± 29 (n = 3)	446 ± 353 (n = 3)
LLL12	STAT3 inhibitor	22 ± 13 (n = 3)	80 ± 2 (n = 3)

Values represent mean ± SD; n, number of independent experiments. Abbreviations: n.a. not annotated; n.d. not determined.

Supplemental Table 6. Variable gene expression Pearson correlations at cluster level

Supplemental Table 7. Variable gene expression Pearson correlations at sub–cluster level

Supplemental Table 8. Fractional contribution of SingleR correlated co–culture cells to query populations

Supplemental Table 9. Cell number at sub–cluster level

Supplemental Table 10. TaqMan Assays

Gene	Assay
ACTA2	Hs00426835_g1
CCN2/CTGF	Hs00170014_m1
CDH1	Hs01023894_m1
COL1A1	Hs00164004_m1
CXCL6	Hs00605742_g1
CXCL8/IL8	Hs00174103_m1
EDN1	Hs00174961_m1
ELN	Hs00355783_m1
FN1	Hs00365052_m1
GPR87	Hs00225057_m1
IL6	Hs00985639_m1
ITGB6	Hs00168458_m1
KRT5	Hs00361185_m1
PDGFA	Hs00964426_m1
PDPN	Hs00366766_m1
TGFB1	Hs00171257_m1
TNF	Hs01113624_g1
TP63	Hs00978340_m1
VIM	Hs00185584_m1
**Reference genes**	**Assay**
18s	Hs99999901_s1

B2M	Hs00984230_m1
GUSB	Hs00939627_m1
HPRT1	Hs02800695_m1
PGK1	Hs00943178_g1
PPIA	Hs04194521_s1
YWHAZ	Hs03044281_g1

Supplemental Table 11. Mapping of Habermann(24) cell identity labels to proposed consensus re–annotation of integrated single–cell transcriptomic human lung cell atlas(30)

**Cell identity labels as used by Habermann, Banovich, and Kropski et al. (24)**	**Proposed consensus hierarchical reference frame of the integrated single–cell transcriptomic atlas of the human lung(30)**
Basal Epithelial Cells	Basal
KRT5-/KRT17+ Epithelial Cells	KRT5-/KRT17+ epithelial
PLIN2+ Fibroblasts	Fibroblasts PLIN2+
HAS1 High Fibroblasts	Subpleural fibroblasts
Myofibroblasts	Myofibroblasts
Smooth Muscle Cells	Smooth Muscle

Supplemental Table 12. R functions with specified arguments

Files

Supplemental File 1. Cell Profiler Pipeline

References

1. Sieber P, Schafer A, Lieberherr R, Le Goff F, Stritt M, Welford RWD, et al. Novel high-throughput myofibroblast assays identify agonists with therapeutic potential in pulmonary fibrosis that act via EP2 and EP4 receptors. *PLoS One.* 2018;13(11):e0207872.

2. Vandesompele J, De Preter K, Pattyn F, Poppe B, Van Roy N, De Paepe A, et al. Accurate normalization of real-time quantitative RT-PCR data by geometric averaging of multiple internal control genes. *Genome Biol.* 2002;3(7):RESEARCH0034.
3. McCarthy DJ, Chen Y, and Smyth GK. Differential expression analysis of multifactor RNA-Seq experiments with respect to biological variation. *Nucleic Acids Res.* 2012;40(10):4288-97.
4. Robinson MD, McCarthy DJ, and Smyth GK. edgeR: a Bioconductor package for differential expression analysis of digital gene expression data. *Bioinformatics.* 2010;26(1):139-40.
5. Futschik ME, and Carlisle B. Noise-robust soft clustering of gene expression time-course data. *J Bioinform Comput Biol.* 2005;3(4):965-88.
6. Kumar L, and M EF. Mfuzz: a software package for soft clustering of microarray data. *Bioinformation.* 2007;2(1):5-7.
7. Conway JR, Lex A, and Gehlenborg N. UpSetR: an R package for the visualization of intersecting sets and their properties. *Bioinformatics.* 2017;33(18):2938-40.
8. Federico A, and Monti S. hypeR: an R package for geneset enrichment workflows. *Bioinformatics.* 2020;36(4):1307-8.
9. Liberzon A, Subramanian A, Pinchback R, Thorvaldsdottir H, Tamayo P, and Mesirov JP. Molecular signatures database (MSigDB) 3.0. *Bioinformatics.* 2011;27(12):1739-40.
10. Subramanian A, Tamayo P, Mootha VK, Mukherjee S, Ebert BL, Gillette MA, et al. Gene set enrichment analysis: a knowledge-based approach for interpreting genome-wide expression profiles. *Proc Natl Acad Sci U S A.* 2005;102(43):15545-50.
11. Chen EY, Tan CM, Kou Y, Duan Q, Wang Z, Meirelles GV, et al. Enrichr: interactive and collaborative HTML5 gene list enrichment analysis tool. *BMC Bioinformatics.* 2013;14:128.
12. Kuleshov MV, Jones MR, Rouillard AD, Fernandez NF, Duan Q, Wang Z, et al. Enrichr: a comprehensive gene set enrichment analysis web server 2016 update. *Nucleic Acids Res.* 2016;44(W1):W90-7.
13. Germain PL, Sonrel A, and Robinson MD. pipeComp, a general framework for the evaluation of computational pipelines, reveals performant single cell RNA-seq preprocessing tools. *Genome Biol.* 2020;21(1):227.
14. Hafemeister C, and Satija R. Normalization and variance stabilization of single-cell RNA-seq data using regularized negative binomial regression. *Genome Biol.* 2019;20(1):296.
15. Tirosh I, Izar B, Prakadan SM, Wadsworth MH, 2nd, Treacy D, Trombetta JJ, et al. Dissecting the multicellular ecosystem of metastatic melanoma by single-cell RNA-seq. *Science.* 2016;352(6282):189-96.
16. Stuart T, Butler A, Hoffman P, Hafemeister C, Papalexi E, Mauck WM, 3rd, et al. Comprehensive Integration of Single-Cell Data. *Cell.* 2019;177(7):1888-902 e21.
17. Zappia L, and Oshlack A. Clustering trees: a visualization for evaluating clusterings at multiple resolutions. *Gigascience.* 2018;7(7).
18. Skinnider MA, Squair JW, Kathe C, Anderson MA, Gautier M, Matson KJE, et al. Cell type prioritization in single-cell data. *Nat Biotechnol.* 2021;39(1):30-4.
19. Finak G, McDavid A, Yajima M, Deng J, Gersuk V, Shalek AK, et al. MAST: a flexible statistical framework for assessing transcriptional changes and characterizing heterogeneity in single-cell RNA sequencing data. *Genome Biol.* 2015;16:278.
20. Street K, Risso D, Fletcher RB, Das D, Ngai J, Yosef N, et al. Slingshot: cell lineage and pseudotime inference for single-cell transcriptomics. *BMC Genomics.* 2018;19(1):477.
21. Qiu X, Mao Q, Tang Y, Wang L, Chawla R, Pliner HA, et al. Reversed graph embedding resolves complex single-cell trajectories. *Nat Methods.* 2017;14(10):979-82.
22. Efremova M, Vento-Tormo M, Teichmann SA, and Vento-Tormo R. CellPhoneDB: inferring cell-cell communication from combined expression of multi-subunit ligand-receptor complexes. *Nat Protoc.* 2020;15(4):1484-506.

23. Shannon P, Markiel A, Ozier O, Baliga NS, Wang JT, Ramage D, et al. Cytoscape: a software environment for integrated models of biomolecular interaction networks. *Genome Res.* 2003;13(11):2498-504.
24. Habermann AC, Gutierrez AJ, Bui LT, Yahn SL, Winters NI, Calvi CL, et al. Single-cell RNA sequencing reveals profibrotic roles of distinct epithelial and mesenchymal lineages in pulmonary fibrosis. *Sci Adv.* 2020;6(28):eaba1972.
25. Aran D, Looney AP, Liu L, Wu E, Fong V, Hsu A, et al. Reference-based analysis of lung single-cell sequencing reveals a transitional profibrotic macrophage. *Nat Immunol.* 2019;20(2):163-72.
26. Tirosh I, Venteicher AS, Hebert C, Escalante LE, Patel AP, Yizhak K, et al. Single-cell RNA-seq supports a developmental hierarchy in human oligodendroglioma. *Nature.* 2016;539(7628):309-13.
27. Wang F, Flanagan J, Su N, Wang LC, Bui S, Nielson A, et al. RNAscope: a novel in situ RNA analysis platform for formalin-fixed, paraffin-embedded tissues. *J Mol Diagn.* 2012;14(1):22-9.
28. Wang H, Su N, Wang LC, Wu X, Bui S, Nielsen A, et al. Quantitative ultrasensitive bright-field RNA in situ hybridization with RNAscope. *Methods Mol Biol.* 2014;1211:201-12.
29. Stritt M, Stalder AK, and Vezzali E. Orbit Image Analysis: An open-source whole slide image analysis tool. *PLoS Comput Biol.* 2020;16(2):e1007313.
30. Sikkema L, Strobl D, Zappia L, Madissoon E, Markov N, Zaragosi L, et al. An integrated cell atlas of the human lung in health and disease. *bioRxiv.* 2022:2022.03.10.483747.
