# Supplementary material for: NF-κB drives epithelial-mesenchymal mechanisms of lung fibrosis in a translational lung cell model
Source: JCI Insight. 2023 Feb 8;8(3):e154719. doi: 10.1172/jci.insight.154719 (PMC9977429; doi:10.1172/jci.insight.154719)
Supplement: Supplemental data set 1 [file jciinsight-8-154719-s047.pdf]

```
CellProfiler Pipeline: http://www.cellprofiler.org
Version:3
DateRevision:20140723174500
GitHash:6c2d896
ModuleCount:14
HasImagePlaneDetails:False

LoadImages:[module_num:1|svn_version:\'Unknown\'|
variable_revision_number:11|show_window:False|notes:\x5B\x5D|
batch_state:array(\x5B\x5D, dtype=uint8)|enabled:True|
wants_pause:False]
    File type to be loaded:individual images
    File selection method:Text-Exact match
    Number of images in each group?:3
    Type the text that the excluded images have in common:Do not use
    Analyze all subfolders within the selected folder?:None
    Input image file location:Default Input Folder\x7C
    Check image sets for unmatched or duplicate files?:Yes
    Group images by metadata?:Yes
    Exclude certain files?:No
    Specify metadata fields to group by:Plate
    Select subfolders to analyze:
    Image count:2
    Text that these images have in common (case-
sensitive):ChNm_DAPIextended
    Position of this image in each group:1
    Extract metadata from where?:File name
    Regular expression that finds metadata in the file name:Batch_(?
P<Batch>\x5B0-9\x5D+)-Plate_(?P<Plate>.*)-Well_(?P<Well>\x5BA-
P\x5D\x5B0-9\x5D{2})-SX_(?P<SiteX>\x5B0-9\x5D)-SY_(?
P<SiteY>\x5B0-9\x5D)-.*-Class_(?P<Class>.*).\x5BA-Za-z0-9\x5D{3}
    Type the regular expression that finds metadata in the subfolder
path:.*\x5B\\\\\\\\/\x5D(?P<Date>.*)\x5B\\\\\\\\/\x5D(?P<Run>.*)$
    Channel count:1
    Group the movie frames?:No
    Grouping method:Interleaved
    Number of channels per group:3
    Load the input as images or objects?:Images
    Name this loaded image:DAPI
    Name this loaded object:Nuclei
    Retain outlines of loaded objects?:No
    Name the outline image:LoadedImageOutlines
    Channel number:1
    Rescale intensities?:No
    Text that these images have in common (case-
sensitive):ChNm_Alexa488
    Position of this image in each group:2
    Extract metadata from where?:File name
    Regular expression that finds metadata in the file name:Batch_(?
P<Batch>\x5B0-9\x5D+)-Plate_(?P<Plate>.*)-Well_(?P<Well>\x5BA-
```

```
P\x5D\x5B0-9\x5D{2})-SX_(?P<SiteX>\x5B0-9\x5D)-SY_(?
P<SiteY>\x5B0-9\x5D)-.*-Class_(?P<Class>.*).\x5BA-Za-z0-9\x5D{3}
    Type the regular expression that finds metadata in the subfolder
path:.*\x5B\\\\\\\\/\x5D(?P<Date>.*)\x5B\\\\\\\\/\x5D(?P<Run>.*)$
    Channel count:1
    Group the movie frames?:No
    Grouping method:Interleaved
    Number of channels per group:3
    Load the input as images or objects?:Images
    Name this loaded image:ACT
    Name this loaded object:Nuclei
    Retain outlines of loaded objects?:No
    Name the outline image:LoadedImageOutlines
    Channel number:1
    Rescale intensities?:No

CorrectIlluminationCalculate:[module_num:2|svn_version:\'Unknown\'|
variable_revision_number:2|show_window:False|notes:\x5B\x5D|
batch_state:array(\x5B\x5D, dtype=uint8)|enabled:True|
wants_pause:False]
    Select the input image:DAPI
    Name the output image:IllumDAPI
    Select how the illumination function is calculated:Background
    Dilate objects in the final averaged image?:No
    Dilation radius:1
    Block size:80
    Rescale the illumination function?:No
    Calculate function for each image individually, or based on all
images?:Each
    Smoothing method:Fit Polynomial
    Method to calculate smoothing filter size:Automatic
    Approximate object size:10
    Smoothing filter size:10
    Retain the averaged image?:No
    Name the averaged image:IllumBlueAvg
    Retain the dilated image?:No
    Name the dilated image:IllumBlueDilated
    Automatically calculate spline parameters?:Yes
    Background mode:auto
    Number of spline points:5
    Background threshold:2
    Image resampling factor:2
    Maximum number of iterations:40
    Residual value for convergence:0.001

CorrectIlluminationCalculate:[module_num:3|svn_version:\'Unknown\'|
variable_revision_number:2|show_window:False|notes:\x5B\x5D|
batch_state:array(\x5B\x5D, dtype=uint8)|enabled:True|
wants_pause:False]
    Select the input image:ACT
```

```
    Name the output image:IllumAct
    Select how the illumination function is calculated:Background
    Dilate objects in the final averaged image?:No
    Dilation radius:1
    Block size:80
    Rescale the illumination function?:No
    Calculate function for each image individually, or based on all
images?:Each
    Smoothing method:Fit Polynomial
    Method to calculate smoothing filter size:Automatic
    Approximate object size:10
    Smoothing filter size:10
    Retain the averaged image?:No
    Name the averaged image:IllumBlueAvg
    Retain the dilated image?:No
    Name the dilated image:IllumBlueDilated
    Automatically calculate spline parameters?:Yes
    Background mode:auto
    Number of spline points:5
    Background threshold:2
    Image resampling factor:2
    Maximum number of iterations:40
    Residual value for convergence:0.001

CorrectIlluminationApply:[module_num:4|svn_version:\'Unknown\'|
variable_revision_number:3|show_window:False|notes:\x5B\x5D|
batch_state:array(\x5B\x5D, dtype=uint8)|enabled:True|
wants_pause:False]
    Select the input image:DAPI
    Name the output image:CorrDAPI
    Select the illumination function:IllumDAPI
    Select how the illumination function is applied:Subtract
    Select the input image:ACT
    Name the output image:CorrACT
    Select the illumination function:IllumAct
    Select how the illumination function is applied:Subtract

MeasureImageQuality:[module_num:5|svn_version:\'Unknown\'|
variable_revision_number:5|show_window:False|notes:\x5B\x5D|
batch_state:array(\x5B\x5D, dtype=uint8)|enabled:True|
wants_pause:False]
    Calculate metrics for which images?:Select...
    Image count:1
    Scale count:1
    Threshold count:1
    Select the images to measure:DAPI
    Include the image rescaling value?:Yes
    Calculate blur metrics?:Yes
    Spatial scale for blur measurements:20
    Calculate saturation metrics?:Yes
```

```
    Calculate intensity metrics?:No
    Calculate thresholds?:No
    Use all thresholding methods?:No
    Select a thresholding method:Otsu
    Typical fraction of the image covered by objects:0.1
    Two-class or three-class thresholding?:Two classes
    Minimize the weighted variance or the entropy?:Weighted variance
    Assign pixels in the middle intensity class to the foreground or
the background?:Foreground

IdentifyPrimaryObjects:[module_num:6|svn_version:\'Unknown\'|
variable_revision_number:10|show_window:False|notes:\x5B\x5D|
batch_state:array(\x5B\x5D, dtype=uint8)|enabled:True|
wants_pause:False]
    Select the input image:CorrDAPI
    Name the primary objects to be identified:Nuclei
    Typical diameter of objects, in pixel units (Min,Max):10,200
    Discard objects outside the diameter range?:Yes
    Try to merge too small objects with nearby larger objects?:No
    Discard objects touching the border of the image?:Yes
    Method to distinguish clumped objects:Intensity
    Method to draw dividing lines between clumped objects:Intensity
    Size of smoothing filter:10
    Suppress local maxima that are closer than this minimum allowed
distance:7
    Speed up by using lower-resolution image to find local maxima?:No
    Name the outline image:PrimaryOutlines
    Fill holes in identified objects?:After both thresholding and
declumping
    Automatically calculate size of smoothing filter for
declumping?:Yes
    Automatically calculate minimum allowed distance between local
maxima?:Yes
    Retain outlines of the identified objects?:No
    Automatically calculate the threshold using the Otsu method?:Yes
    Enter Laplacian of Gaussian threshold:0.5
    Automatically calculate the size of objects for the Laplacian of
Gaussian filter?:Yes
    Enter LoG filter diameter:5
    Handling of objects if excessive number of objects
identified:Continue
    Maximum number of objects:2000
    Threshold setting version:1
    Threshold strategy:Global
    Thresholding method:Otsu
    Select the smoothing method for thresholding:Automatic
    Threshold smoothing scale:1
    Threshold correction factor:0.5
    Lower and upper bounds on threshold:0.05,0.1
    Approximate fraction of image covered by objects?:0.01
```

```
    Manual threshold:0.0
    Select the measurement to threshold with:None
    Select binary image:None
    Masking objects:From image
    Two-class or three-class thresholding?:Two classes
    Minimize the weighted variance or the entropy?:Weighted variance
    Assign pixels in the middle intensity class to the foreground or
the background?:Foreground
    Method to calculate adaptive window size:Image size
    Size of adaptive window:10

IdentifySecondaryObjects:[module_num:7|svn_version:\'Unknown\'|
variable_revision_number:9|show_window:False|notes:\x5B\x5D|
batch_state:array(\x5B\x5D, dtype=uint8)|enabled:True|
wants_pause:True]
    Select the input objects:Nuclei
    Name the objects to be identified:ACTobj
    Select the method to identify the secondary objects:Propagation
    Select the input image:CorrACT
    Number of pixels by which to expand the primary objects:10
    Regularization factor:0.05
    Name the outline image:SecondaryOutlines
    Retain outlines of the identified secondary objects?:No
    Discard secondary objects touching the border of the image?:No
    Discard the associated primary objects?:No
    Name the new primary objects:FilteredNuclei
    Retain outlines of the new primary objects?:No
    Name the new primary object outlines:FilteredNucleiOutlines
    Fill holes in identified objects?:Yes
    Threshold setting version:1
    Threshold strategy:Global
    Thresholding method:RobustBackground
    Select the smoothing method for thresholding:No smoothing
    Threshold smoothing scale:1
    Threshold correction factor:1
    Lower and upper bounds on threshold:0.01,1
    Approximate fraction of image covered by objects?:0.01
    Manual threshold:0.0
    Select the measurement to threshold with:None
    Select binary image:None
    Masking objects:From image
    Two-class or three-class thresholding?:Two classes
    Minimize the weighted variance or the entropy?:Weighted variance
    Assign pixels in the middle intensity class to the foreground or
the background?:Foreground
    Method to calculate adaptive window size:Image size
    Size of adaptive window:10

MeasureObjectSizeShape:[module_num:8|svn_version:\'Unknown\'|
variable_revision_number:1|show_window:False|notes:\x5B\x5D|
```

```
batch_state:array(\x5B\x5D, dtype=uint8)|enabled:True|
wants_pause:True]
    Select objects to measure:Nuclei
    Select objects to measure:ACTobj
    Calculate the Zernike features?:Yes

MeasureTexture:[module_num:9|svn_version:\'Unknown\'|
variable_revision_number:3|show_window:False|notes:\x5B\x5D|
batch_state:array(\x5B\x5D, dtype=uint8)|enabled:True|
wants_pause:False]
    Hidden:2
    Hidden:2
    Hidden:1
    Select an image to measure:IllumAct
    Select an image to measure:CorrACT
    Select objects to measure:Nuclei
    Select objects to measure:ACTobj
    Texture scale to measure:3
    Angles to measure:Horizontal
    Measure Gabor features?:Yes
    Number of angles to compute for Gabor:4

MeasureGranularity:[module_num:10|svn_version:\'Unknown\'|
variable_revision_number:3|show_window:False|notes:\x5B\x5D|
batch_state:array(\x5B\x5D, dtype=uint8)|enabled:True|
wants_pause:False]
    Image count:1
    Object count:1
    Select an image to measure:CorrACT
    Subsampling factor for granularity measurements:0.25
    Subsampling factor for background reduction:0.25
    Radius of structuring element:10
    Range of the granular spectrum:16
    Select objects to measure:ACTobj

MeasureObjectIntensity:[module_num:11|svn_version:\'Unknown\'|
variable_revision_number:3|show_window:False|notes:\x5B\x5D|
batch_state:array(\x5B\x5D, dtype=uint8)|enabled:True|
wants_pause:False]
    Hidden:1
    Select an image to measure:CorrACT
    Select objects to measure:ACTobj

MeasureObjectNeighbors:[module_num:12|svn_version:\'Unknown\'|
variable_revision_number:2|show_window:False|notes:\x5B\x5D|
batch_state:array(\x5B\x5D, dtype=uint8)|enabled:True|
wants_pause:False]
    Select objects to measure:Nuclei
    Select neighboring objects to measure:Nuclei
    Method to determine neighbors:Expand until adjacent
```

```
    Neighbor distance:5
    Retain the image of objects colored by numbers of neighbors?:No
    Name the output image:ObjectNeighborCount
    Select colormap:Default
    Retain the image of objects colored by percent of touching
pixels?:No
    Name the output image:PercentTouching
    Select a colormap:Default

MeasureObjectRadialDistribution:[module_num:13|svn_version:
\'Unknown\'|variable_revision_number:3|show_window:False|notes:
\x5B\x5D|batch_state:array(\x5B\x5D, dtype=uint8)|enabled:True|
wants_pause:False]
    Hidden:1
    Hidden:1
    Hidden:1
    Select an image to measure:CorrACT
    Select objects to measure:ACTobj
    Object to use as center?:Centers of other objects
    Select objects to use as centers:Nuclei
    Scale the bins?:Yes
    Number of bins:4
    Maximum radius:100

ExportToSpreadsheet:[module_num:14|svn_version:\'Unknown\'|
variable_revision_number:11|show_window:False|notes:\x5B\x5D|
batch_state:array(\x5B\x5D, dtype=uint8)|enabled:True|
wants_pause:False]
    Select the column delimiter:Comma (",")
    Add image metadata columns to your object data file?:Yes
    Limit output to a size that is allowed in Excel?:No
    Select the measurements to export:Yes
    Calculate the per-image mean values for object measurements?:Yes
    Calculate the per-image median values for object measurements?:No
    Calculate the per-image standard deviation values for object
measurements?:No
    Output file location:Default Output Folder\x7CNone
    Create a GenePattern GCT file?:No
    Select source of sample row name:Metadata
    Select the image to use as the identifier:None
    Select the metadata to use as the identifier:None
    Export all measurement types?:Yes
    Press button to select measurements to
export:Image\x7CCount_Nuclei,Image\x7CCount_ACTobj,Image\x7CImageQuali
ty_LocalFocusScore_DAPI_20,Image\x7CImageQuality_PercentMaximal_DAPI,I
mage\x7CImageQuality_PowerLogLogSlope_DAPI,Image\x7CImageQuality_Scali
ng_DAPI,Image\x7CImageQuality_PercentMinimal_DAPI,Image\x7CImageQualit
y_FocusScore_DAPI,Image\x7CImageQuality_Correlation_DAPI_20,Image\x7CW
idth_DAPI,Image\x7CWidth_ACT,Image\x7CFileName_DAPI,Image\x7CFileName_
ACT,Image\x7CURL_DAPI,Image\x7CURL_ACT,Image\x7CTexture_DifferenceEntr
```

opy_CorrACT_3_0,Image\x7CTexture_InfoMeas2_CorrACT_3_0,Image\x7CTextur
e_InfoMeas1_CorrACT_3_0,Image\x7CTexture_SumVariance_CorrACT_3_0,Image
\x7CTexture_Gabor_CorrACT_3,Image\x7CTexture_AngularSecondMoment_CorrA
CT_3_0,Image\x7CTexture_DifferenceVariance_CorrACT_3_0,Image\x7CTextur
e_Entropy_CorrACT_3_0,Image\x7CTexture_Correlation_CorrACT_3_0,Image\x
7CTexture_SumAverage_CorrACT_3_0,Image\x7CTexture_Variance_CorrACT_3_0
,Image\x7CTexture_SumEntropy_CorrACT_3_0,Image\x7CTexture_InverseDiffe
renceMoment_CorrACT_3_0,Image\x7CTexture_Contrast_CorrACT_3_0,Image\x7
CHeight_DAPI,Image\x7CHeight_ACT,Image\x7CScaling_DAPI,Image\x7CScalin
g_ACT,Image\x7CPathName_DAPI,Image\x7CPathName_ACT,Image\x7CGranularit
y_11_CorrACT,Image\x7CGranularity_10_CorrACT,Image\x7CGranularity_13_C
orrACT,Image\x7CGranularity_12_CorrACT,Image\x7CGranularity_14_CorrACT
,Image\x7CGranularity_16_CorrACT,Image\x7CGranularity_1_CorrACT,Image\
x7CGranularity_3_CorrACT,Image\x7CGranularity_2_CorrACT,Image\x7CGranu
larity_5_CorrACT,Image\x7CGranularity_8_CorrACT,Image\x7CGranularity_7
_CorrACT,Image\x7CGranularity_6_CorrACT,Image\x7CGranularity_9_CorrACT
,Image\x7CGranularity_15_CorrACT,Image\x7CGranularity_4_CorrACT,Image\
x7CThreshold_OrigThreshold_Nuclei,Image\x7CThreshold_OrigThreshold_ACT
obj,Image\x7CThreshold_SumOfEntropies_Nuclei,Image\x7CThreshold_SumOfE
ntropies_ACTobj,Image\x7CThreshold_WeightedVariance_Nuclei,Image\x7CTh
reshold_WeightedVariance_ACTobj,Image\x7CThreshold_FinalThreshold_Nucl
ei,Image\x7CThreshold_FinalThreshold_ACTobj,Image\x7CGroup_Index,Image
\x7CGroup_Number,Image\x7CMD5Digest_DAPI,Image\x7CMD5Digest_ACT,Image\
x7CMetadata_Plate,Image\x7CMetadata_SiteX,Image\x7CMetadata_SiteY,Imag
e\x7CMetadata_Well,Image\x7CMetadata_Batch,Image\x7CMetadata_Class,Nuc
lei\x7CNeighbors_SecondClosestDistance_Expanded,Nuclei\x7CNeighbors_Fi
rstClosestDistance_Expanded,Nuclei\x7CNeighbors_FirstClosestObjectNumb
er_Expanded,Nuclei\x7CNeighbors_SecondClosestObjectNumber_Expanded,Nuc
lei\x7CNeighbors_PercentTouching_Expanded,Nuclei\x7CNeighbors_NumberOf
Neighbors_Expanded,Nuclei\x7CNeighbors_AngleBetweenNeighbors_Expanded,
Nuclei\x7CNumber_Object_Number,Nuclei\x7CTexture_DifferenceVariance_Co
rrACT_3_0,Nuclei\x7CTexture_InfoMeas1_CorrACT_3_0,Nuclei\x7CTexture_Su
mVariance_CorrACT_3_0,Nuclei\x7CTexture_Gabor_CorrACT_3,Nuclei\x7CText
ure_AngularSecondMoment_CorrACT_3_0,Nuclei\x7CTexture_Correlation_Corr
ACT_3_0,Nuclei\x7CTexture_Entropy_CorrACT_3_0,Nuclei\x7CTexture_Differ
enceEntropy_CorrACT_3_0,Nuclei\x7CTexture_SumAverage_CorrACT_3_0,Nucle
i\x7CTexture_Variance_CorrACT_3_0,Nuclei\x7CTexture_InverseDifferenceM
oment_CorrACT_3_0,Nuclei\x7CTexture_SumEntropy_CorrACT_3_0,Nuclei\x7CT
exture_Contrast_CorrACT_3_0,Nuclei\x7CTexture_InfoMeas2_CorrACT_3_0,Nu
clei\x7CLocation_Center_Y,Nuclei\x7CLocation_Center_X,Nuclei\x7CAreaSh
ape_Perimeter,Nuclei\x7CAreaShape_FormFactor,Nuclei\x7CAreaShape_Solid
ity,Nuclei\x7CAreaShape_Center_Y,Nuclei\x7CAreaShape_Center_X,Nuclei\x
7CAreaShape_MaxFeretDiameter,Nuclei\x7CAreaShape_MinFeretDiameter,Nucl
ei\x7CAreaShape_Area,Nuclei\x7CAreaShape_EulerNumber,Nuclei\x7CAreaSha
pe_Zernike_1_1,Nuclei\x7CAreaShape_Zernike_0_0,Nuclei\x7CAreaShape_Zer
nike_3_1,Nuclei\x7CAreaShape_Zernike_3_3,Nuclei\x7CAreaShape_Zernike_2
_0,Nuclei\x7CAreaShape_Zernike_2_2,Nuclei\x7CAreaShape_Zernike_5_1,Nuc
lei\x7CAreaShape_Zernike_5_3,Nuclei\x7CAreaShape_Zernike_5_5,Nuclei\x7
CAreaShape_Zernike_4_0,Nuclei\x7CAreaShape_Zernike_4_2,Nuclei\x7CAreaS
hape_Zernike_4_4,Nuclei\x7CAreaShape_Zernike_7_1,Nuclei\x7CAreaShape_Z

ernike_7_3,Nuclei\x7CAreaShape_Zernike_7_5,Nuclei\x7CAreaShape_Zernike
_7_7,Nuclei\x7CAreaShape_Zernike_6_0,Nuclei\x7CAreaShape_Zernike_6_2,N
uclei\x7CAreaShape_Zernike_6_4,Nuclei\x7CAreaShape_Zernike_6_6,Nuclei\
x7CAreaShape_Zernike_9_1,Nuclei\x7CAreaShape_Zernike_9_3,Nuclei\x7CAre
aShape_Zernike_9_5,Nuclei\x7CAreaShape_Zernike_9_7,Nuclei\x7CAreaShape
_Zernike_9_9,Nuclei\x7CAreaShape_Zernike_8_0,Nuclei\x7CAreaShape_Zerni
ke_8_2,Nuclei\x7CAreaShape_Zernike_8_4,Nuclei\x7CAreaShape_Zernike_8_6
,Nuclei\x7CAreaShape_Zernike_8_8,Nuclei\x7CAreaShape_Eccentricity,Nucl
ei\x7CAreaShape_Compactness,Nuclei\x7CAreaShape_Extent,Nuclei\x7CAreaS
hape_Orientation,Nuclei\x7CAreaShape_MedianRadius,Nuclei\x7CAreaShape_
MaximumRadius,Nuclei\x7CAreaShape_MinorAxisLength,Nuclei\x7CAreaShape_
MajorAxisLength,Nuclei\x7CAreaShape_MeanRadius,Nuclei\x7CChildren_ACTo
bj_Count,ACTobj\x7CParent_Nuclei,ACTobj\x7CGranularity_11_CorrACT,ACTo
bj\x7CGranularity_10_CorrACT,ACTobj\x7CGranularity_13_CorrACT,ACTobj\x
7CGranularity_12_CorrACT,ACTobj\x7CGranularity_15_CorrACT,ACTobj\x7CGr
anularity_14_CorrACT,ACTobj\x7CGranularity_16_CorrACT,ACTobj\x7CGranul
arity_1_CorrACT,ACTobj\x7CGranularity_3_CorrACT,ACTobj\x7CGranularity_
2_CorrACT,ACTobj\x7CGranularity_5_CorrACT,ACTobj\x7CGranularity_4_Corr
ACT,ACTobj\x7CGranularity_7_CorrACT,ACTobj\x7CGranularity_6_CorrACT,AC
Tobj\x7CGranularity_9_CorrACT,ACTobj\x7CGranularity_8_CorrACT,ACTobj\x
7CNumber_Object_Number,ACTobj\x7CTexture_InfoMeas2_CorrACT_3_0,ACTobj\
x7CTexture_InfoMeas1_CorrACT_3_0,ACTobj\x7CTexture_SumVariance_CorrACT
_3_0,ACTobj\x7CTexture_Gabor_CorrACT_3,ACTobj\x7CTexture_DifferenceEnt
ropy_CorrACT_3_0,ACTobj\x7CTexture_Correlation_CorrACT_3_0,ACTobj\x7CT
exture_SumAverage_CorrACT_3_0,ACTobj\x7CTexture_Entropy_CorrACT_3_0,AC
Tobj\x7CTexture_AngularSecondMoment_CorrACT_3_0,ACTobj\x7CTexture_Diff
erenceVariance_CorrACT_3_0,ACTobj\x7CTexture_Variance_CorrACT_3_0,ACTo
bj\x7CTexture_SumEntropy_CorrACT_3_0,ACTobj\x7CTexture_InverseDifferen
ceMoment_CorrACT_3_0,ACTobj\x7CTexture_Contrast_CorrACT_3_0,ACTobj\x7C
Intensity_MassDisplacement_CorrACT,ACTobj\x7CIntensity_MinIntensity_Co
rrACT,ACTobj\x7CIntensity_StdIntensity_CorrACT,ACTobj\x7CIntensity_Int
egratedIntensityEdge_CorrACT,ACTobj\x7CIntensity_UpperQuartileIntensit
y_CorrACT,ACTobj\x7CIntensity_LowerQuartileIntensity_CorrACT,ACTobj\x7
CIntensity_MinIntensityEdge_CorrACT,ACTobj\x7CIntensity_MADIntensity_C
orrACT,ACTobj\x7CIntensity_IntegratedIntensity_CorrACT,ACTobj\x7CInten
sity_MeanIntensityEdge_CorrACT,ACTobj\x7CIntensity_MaxIntensity_CorrAC
T,ACTobj\x7CIntensity_MedianIntensity_CorrACT,ACTobj\x7CIntensity_Mean
Intensity_CorrACT,ACTobj\x7CIntensity_StdIntensityEdge_CorrACT,ACTobj\
x7CIntensity_MaxIntensityEdge_CorrACT,ACTobj\x7CLocation_MaxIntensity_
Y_CorrACT,ACTobj\x7CLocation_MaxIntensity_X_CorrACT,ACTobj\x7CLocation
_Center_Y,ACTobj\x7CLocation_Center_X,ACTobj\x7CLocation_CenterMassInt
ensity_Y_CorrACT,ACTobj\x7CLocation_CenterMassIntensity_X_CorrACT,ACTo
bj\x7CAreaShape_Perimeter,ACTobj\x7CAreaShape_FormFactor,ACTobj\x7CAre
aShape_MeanRadius,ACTobj\x7CAreaShape_Orientation,ACTobj\x7CAreaShape_
Area,ACTobj\x7CAreaShape_MinFeretDiameter,ACTobj\x7CAreaShape_Solidity
,ACTobj\x7CAreaShape_MaxFeretDiameter,ACTobj\x7CAreaShape_EulerNumber,
ACTobj\x7CAreaShape_Zernike_1_1,ACTobj\x7CAreaShape_Zernike_0_0,ACTobj
\x7CAreaShape_Zernike_3_1,ACTobj\x7CAreaShape_Zernike_3_3,ACTobj\x7CAr
eaShape_Zernike_2_0,ACTobj\x7CAreaShape_Zernike_2_2,ACTobj\x7CAreaShap
e_Zernike_5_1,ACTobj\x7CAreaShape_Zernike_5_5,ACTobj\x7CAreaShape_Zern

```
ike_5_3,ACTobj\x7CAreaShape_Zernike_4_0,ACTobj\x7CAreaShape_Zernike_4_
2,ACTobj\x7CAreaShape_Zernike_4_4,ACTobj\x7CAreaShape_Zernike_7_1,ACTo
bj\x7CAreaShape_Zernike_7_5,ACTobj\x7CAreaShape_Zernike_7_3,ACTobj\x7C
AreaShape_Zernike_7_7,ACTobj\x7CAreaShape_Zernike_6_0,ACTobj\x7CAreaSh
ape_Zernike_6_2,ACTobj\x7CAreaShape_Zernike_6_4,ACTobj\x7CAreaShape_Ze
rnike_6_6,ACTobj\x7CAreaShape_Zernike_9_1,ACTobj\x7CAreaShape_Zernike_
9_3,ACTobj\x7CAreaShape_Zernike_9_5,ACTobj\x7CAreaShape_Zernike_9_7,AC
Tobj\x7CAreaShape_Zernike_9_9,ACTobj\x7CAreaShape_Zernike_8_0,ACTobj\x
7CAreaShape_Zernike_8_2,ACTobj\x7CAreaShape_Zernike_8_4,ACTobj\x7CArea
Shape_Zernike_8_6,ACTobj\x7CAreaShape_Zernike_8_8,ACTobj\x7CAreaShape_
MedianRadius,ACTobj\x7CAreaShape_Compactness,ACTobj\x7CAreaShape_Exten
t,ACTobj\x7CAreaShape_Eccentricity,ACTobj\x7CAreaShape_MaximumRadius,A
CTobj\x7CAreaShape_MinorAxisLength,ACTobj\x7CAreaShape_MajorAxisLength
,ACTobj\x7CAreaShape_Center_Y,ACTobj\x7CAreaShape_Center_X,ACTobj\x7CR
adialDistribution_RadialCV_CorrACT_3of4,ACTobj\x7CRadialDistribution_R
adialCV_CorrACT_4of4,ACTobj\x7CRadialDistribution_RadialCV_CorrACT_1of
4,ACTobj\x7CRadialDistribution_RadialCV_CorrACT_2of4,ACTobj\x7CRadialD
istribution_FracAtD_CorrACT_3of4,ACTobj\x7CRadialDistribution_FracAtD_
CorrACT_4of4,ACTobj\x7CRadialDistribution_FracAtD_CorrACT_1of4,ACTobj\
x7CRadialDistribution_FracAtD_CorrACT_2of4,ACTobj\x7CRadialDistributio
n_MeanFrac_CorrACT_3of4,ACTobj\x7CRadialDistribution_MeanFrac_CorrACT_
4of4,ACTobj\x7CRadialDistribution_MeanFrac_CorrACT_1of4,ACTobj\x7CRadi
alDistribution_MeanFrac_CorrACT_2of4
    Representation of Nan/Inf:NaN
    Add a prefix to file names?:No
    Filename prefix\x3A:MyExpt_
    Overwrite without warning?:Yes
    Data to export:Do not use
    Combine these object measurements with those of the previous
object?:No
    File name:DATA.csv
    Use the object name for the file name?:Yes
```
